# An essential signaling function of cytoplasmic NELFB is independent of RNA polymerase II pausing

**DOI:** 10.1016/j.jbc.2023.105259

**Published:** 2023-09-17

**Authors:** Haihui Pan, Xiaolong Cheng, Pedro Felipe Gardeazábal Rodríguez, Xiaowen Zhang, Inhee Chung, Victor X. Jin, Wei Li, Yanfen Hu, Rong Li

**Affiliations:** 1 Department of Biochemistry & Molecular Medicine, School of Medicine & Health Sciences, The George Washington University, Washington, District of Columbia, USA; 2 Department of Genomics & Precision Medicine, School of Medicine & Health Sciences, The George Washington University, Washington, District of Columbia, USA; 3 Center for Genetic Medicine Research, Children's National Hospital, Washington, District of Columbia, USA; 4 Department of Anatomy & Cell Biology, School of Medicine & Health Sciences, The George Washington University, Washington, District of Columbia, USA; 5 Institute of Health Equity and Cancer Center, The Medical College of Wisconsin, Milwaukee, Wisconsin, USA

**Keywords:** NELF, NELFB, Pol II pausing, PI3K/AKT, APEX2, AID, IAA induced rapid degradation

## Abstract

The four-subunit negative elongation factor (NELF) complex mediates RNA polymerase II (Pol II) pausing at promoter-proximal regions. Ablation of individual NELF subunits destabilizes the NELF complex and causes cell lethality, leading to the prevailing concept that NELF-mediated Pol II pausing is essential for cell proliferation. Using separation-of-function mutations, we show here that NELFB function in cell proliferation can be uncoupled from that in Pol II pausing. NELFB mutants sequestered in the cytoplasm and deprived of NELF nuclear function still support cell proliferation and part of the NELFB-dependent transcriptome. Mechanistically, cytoplasmic NELFB physically and functionally interacts with prosurvival signaling kinases, most notably phosphatidylinositol-3-kinase/AKT. Ectopic expression of membrane-tethered phosphatidylinositol-3-kinase/AKT partially bypasses the role of NELFB in cell proliferation, but not Pol II occupancy. Together, these data expand the current understanding of the physiological impact of Pol II pausing and underscore the multiplicity of the biological functions of individual NELF subunits.

Promoter-proximal RNA polymerase II (Pol II) pausing is a widespread phenomenon across the whole genome of most metazoans (1). Following transcription initiation, Pol II is paused after transcribing 20 ∼ 60 nucleotides (2, 3). Pol II pausing is thought to be a crucial regulatory mechanism by which genes are activated transcriptionally in response to environmental and developmental signals (4). The negative elongation factor (NELF) complex enforces and maintains Pol II pausing, in collaboration with the DRB sensitivity-inducing factor (5, 6, 7, 8, 9). NELF consists of four protein subunits: NELFA, NELFB, NELFC, and NELFE (7). Depletion of any NELF subunit results in destabilization of the remaining subunits (10, 11). Upon phosphorylation by the positive transcription elongation factor, NELF dissociates from paused Pol II and thus allows resumption of transcription elongation (12). Recent cryo-EM work suggests that NELF restrains the movement of Pol II *via* multiple mechanisms: it directly restricts polymerase movement by binding along the Pol II funnel, interferes with nucleotide triphosphate diffusion and addition, and prevents both transcription elongation factor IIS and Pol II-associated factor from binding to Pol II (13). Consistent with the structural and biochemical knowledge of NELF, its depletion in cultured cells reduces the occupancy of promoter-proximal Pol II (14, 15, 16). However, a recent study shows that the acute loss of promoter-proximal Pol II is not immediately accompanied by a corresponding increase in Pol II signals within the gene body (GB) (15). This suggests that NELF may not simply serve as an elongation inhibitor but rather a checkpoint to retain the local transcription machinery and prevent mRNA from premature termination (4).

In addition to NELF’s activity in Pol II pausing, its cellular functions have been explored extensively *via* genetic ablation of various NELF subunits, especially NELFB. NELFB is essential for cultured cell proliferation and embryonic stem cell differentiation (17, 18, 19). In the context of adult tissue development, NELFB is known to play pivotal roles in mammary ductal morphogenesis during puberty (20, 21), uterine decidual development during pregnancy (22), muscle stem cell regeneration (23), and T lymphocyte functions in antitumor immunity (24). NELFB depletion in postmitotic cardiomyocytes causes tissue dysfunction and cardiomyopathy (25). Cell- and tissue-specific NELFB inactivation is often associated with genome-wide reduction of promoter-proximal Pol II pausing and disruption of context-dependent transcription programs (18, 25, 26, 27, 28). However, it remains unclear how global attenuation of Pol II pausing could dampen transcription of a distinct set of genes under a given physiological condition. More mechanistic probing is warranted to fully elucidate the molecular basis of NELF’s biological functions.

Aside from their collective contributions to the functionality of the NELF complex in Pol II pausing, individual NELF subunits have been implicated in disparate cellular functions that sometimes seem incongruent with the canonical NELF activity in Pol II pausing. For instance, NELFA, unlike the other NELF subunits, is a central maternal factor for 2-cell stage–specific gene expression (29). In a separate study, NELFB was shown to physically shuttle tropomyosin receptor kinase C to mitochondria and promotes tropomyosin receptor kinase C–induced apoptosis (30, 31). Because stability of the four NELF subunits is mutually dependent, depletion of one subunit results in reduced levels for the remaining subunits (10, 32, 33). Therefore, it is technically difficult to unequivocally ascribe a biological effect of protein depletion to a specific NELF subunit. For the same reason, it is also challenging to distinguish NELF-dependent from NELF-independent activities by whole-gene deletion. Interrogation of the potential multifaceted nature of NELF molecular activities may require more precise gene-dissecting tools such as separation-of-function mutagenesis, which has been used successfully to demonstrate multiplicity of other biological molecules (34, 35, 36, 37).

Here, we generated a panel of NELFB mutants (MTs) and subjected them to functional assays including cell proliferation, transcription, and Pol II pausing in immortalized mouse embryonic fibroblasts (MEFs) (17). Unexpectedly, the NELF complex’s integrity and its Pol II pausing activity are dispensable for NELFB-dependent transcriptomics and cell proliferation. To interrogate the Pol II pausing–independent function of NELFB, we found that NELFB physically and functionally interacts with prosurvival kinases including phosphatidylinositol-3-kinase (PI3K)/AKT. Interestingly, ectopic expression of membrane-tethered PI3K/AKT kinases partially bypasses the need of NELFB for cell proliferation, but not Pol II occupancy. Altogether, our molecular study not only separates NELFB’s essential function from its Pol II pausing activity but also uncovers a previously unrecognized role of cytoplasmic NELFB in prosurvival signaling.

## Results

### Integrity of the NELF complex is not required for NELFB-dependent cell proliferation

**Figure 1:**
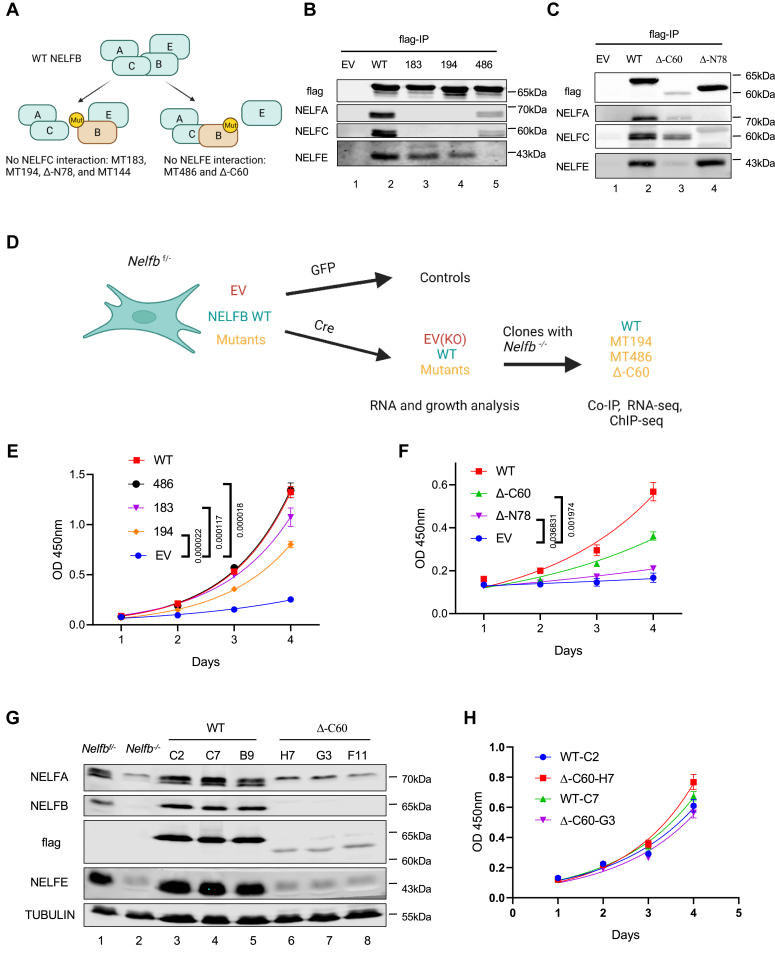
**Integrity of the NELF complex is not required for NELFB-dependent cell proliferation**. *A*, site-specific mutagenesis to disrupt NELFB interactions with other NELF subunits. *B* and *C*, Flag coimmunoprecipitation (co-IP) using HEK293T cells transfected with an empty vector (EV) or flag-tagged mouse NELFB WT and mutants, followed by Western blotting of various NELF subunits. Representative image from three independent experiments was shown. *D*, schematic illustration of NELFB mutant analysis in conditional *Nelfb* KO MEFs. *E* and *F*, stable *Nelfb^f/-^* cell lines with EV, WT, or mutant NELFB were infected with Adeno-Cre for 7 days. Cell numbers were measured daily for four consecutive days using Cell Counting Kit-8 (CCK8). Representative experiment was shown from three independent experiments. Exact *p* values for the last time point are indicated. *G*, Western blotting of different NELF subunits in *Nelfb^−/−^* clones with ectopic WT and DC60. *Nelfb^f/-^* and *Nelfb^−/−^* cell lysates were used as reference. A total of six independent clones for both WT and D-C60 were analyzed and the results of three WT and D-C60 clones were shown. *H*, cell growth of the WT and Δ-C60 clones were measured for four consecutive days using CCK8. Statistical analysis was performed using multiple *t* test. Exact *p* values for the last time point are indicated. MEF, mouse embryonic fibroblast; NELF, negative elongation factor.

NELFB is defined as a common essential gene in the Cancer Dependency Map Project (Fig. S1, *A* and *B*) (38). However, it is not clear whether its essential function depends on its interactions with the other NELF subunits. Based on the configuration of individual NELF subunits in the NELF complex, we conducted several approaches of NELFB mutagenesis to identify mutations that disrupt its binding to either NELFA/NELFC or NELFE (Fig. 1*A*). First, a previous crosslinking study identified several NELFB lysine residues in close proximity with the neighboring NELF subunits (39). We therefore generated a series of alanine-substitution MTs encompassing these lysine residues in NELFB (from XKX to AAA, Table S1). We assessed the affinity of these NELFB MTs for the other NELF subunits by flag-tagged coimmunoprecipitation (co-IP). Out of the 11 alanine–substitution MTs analyzed in both human HEK293T cells and MEFs, MT183 and MT194 completely lost their interactions with NELFA and NELFC while retaining intact binding to NELFE (Figs. 1*B* and S1, *C*–*E*). Conversely, MT486 failed to bind to NELFE but retained at least its partial affinity for NELFA and NELFC (Figs. 1*B* and S1, *C*–*E*). In addition to these alanine-substitution MTs, we also generated amino (N)- and carboxyl (C)-terminal truncated MTs of NELFB. The N-terminal truncation Δ-N78 was able to bind to NELFE, but not NELFA or NELFC (Fig. 1*C*). On the other hand, the C-terminal truncation Δ-C60 was co-IPed with NELFA and NELFC, but not NELFE (Fig. 1*C*). In the last mutational approach, we targeted the LXXLL sequence, a well-known motif that is critical for protein interactions (40). Of the three LXXLL-targeting MTs of NELFB, MT144 failed to bind to NELFC (Fig. S1*C*). Thus, as summarized in Figure 1*A*, we generated a panel of NELFB MTs with the crippled ability of binding to specific NELF subunits.

Next, we ectopically expressed WT and various NELF complex–defective NELFB MTs (MT183, MT194, MT486, Δ-N78, and Δ-C60) in *Nelfb*^*f/-*^ MEFs, which contain a null and a floxed *Nelfb* allele. We then examined the ability of ectopic WT and MTs to support cell proliferation upon Cre-mediated deletion of the endogenous floxed *Nelfb* allele (Fig. 1*D*). As expected, these NELFB MTs were not able to stabilize the other NELF subunits in MEFs depleted of endogenous NELFB (Fig. S1, *F* and *G*). Surprisingly, most of these MTs, except for Δ-N78, largely retained the ability of WT NELFB to support cell proliferation (Fig. 1, *E* and *F*). In particular, MT486, which is devoid of its NELFE-binding ability, is indistinguishable from the WT protein in rescuing the proliferative defect of *Nelfb*^*−/−*^ cells (Fig. 1*E*). Following infection of MEFs with the Cre-expressing virus, we were able to isolate single *Nelfb*^*−/−*^ clones expressing ectopic NELFB MTs, including MT183, MT194, MT486, and Δ-C60 (Figs. S1, *H* and 1*G*). Stable passaging of these clones as homogeneous cell populations further confirms the ability of the NELFB MTs to support long-term cell growth.

Stable clones of Δ-C60–expressing *Nelfb*^*−/−*^ cells exhibited significantly lower levels of NELF subunits *versus* the WT clones (compare lanes 3–5 and 6–8 in Fig. 1*G*). Nevertheless, these MT cell clones proliferated equally well compared to their WT counterparts (Fig. 1*H*). To determine whether the same was true for human NELFB, we used CRISPR-Cas9 in human breast cancer cells MDA-MB-231 to disrupt the corresponding C-terminal portion of human NELFB (hNELFB), which is over 90% identical to its mouse counterpart (Fig. S2, *A* and *B*). Although the gene editing significantly destabilized the truncated hNELFB (Δ-C60) and the other hNELF subunits (Fig. S2, *C* and *D*), the resulting MDA-MB-231 cell clones proliferated as robustly as the WT control (Fig. S2*E*). Collectively, our mutational study strongly suggests that an intact NELF complex is dispensable for NELFB-dependent cell proliferation.

### NELFB-dependent Pol II pausing and cell proliferation are functionally separable

**Figure 2:**
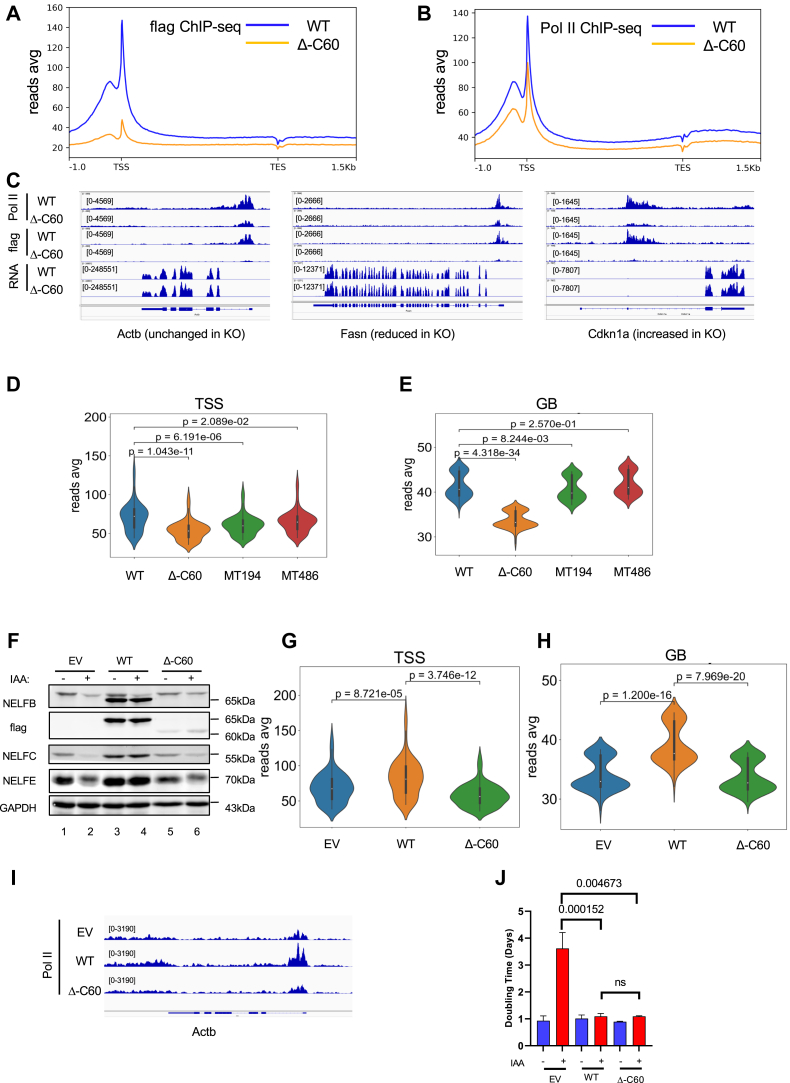
**NELFB-dependent Pol II pausing and cell proliferation are functionally separable**. *A* and *B*, averaged metagene profiles of flag-NELFB (*A*) and Pol II (*B*) chromatin occupancy in WT and Δ-C60 cells. ChIP-seq experiments were performed with two (*A*) and four (*B*) independent biological replicates. The analyzed region is from −1kb upstream of transcription start sites (TSS) to +1.5kb downstream of transcription end sites (TES). *C*, representative IGV profiles of *Actb* (not dependent on NELFB), *Fasn* (NELFB-upregulated), and *Cdkn1a* (NELFB-downregulated). Pol II ChIP-seq, Flag-NELFB ChIP-seq, and RNA-seq are averages of four, two, and three independent clones, respectively. *D* and *E*, *violin plots* showing distributions of Pol II ChIP-seq reads at the TSS (−0.5 to +0.5kb, *D*) and gene body (GB) (+0.5 to +2.5 kb, *E*) in WT and mutant MEFs. Pol II ChIP-seq were from four independent clones of WT and Δ-C60 and two independent clones of MT194 and MT486. *F*, stable NELFB–AID cell lines that expressed EV, WT NELFB, or D-C60 were treated with PBS or 0.5 mM auxin (indole-3-acetic acid, IAA) for 6 h. Representative Western blotting of different NELF subunits from three independent experiments is shown. *G* and *H*, *violin plot* distributions of Pol II ChIP-seq reads at the TSS (−0.5 to +0.5kb, *G*) and GB (+0.5 to +2.5 kb, *H*) in IAA-treated NELFB-AID cells that stably expressed EV, WT, or Δ-C60. Pol II ChIP-seq was from two independent experiments. *I*, representative IGV profiles of *Actb*. Pol II ChIP-seq was averaged from two independent experiments. *J*, stable NELFB–AID cell lines that expressed EV, WT, or Δ-C60 were treated with PBS or 0.5 mM IAA continuously. Cell proliferation was measured for four consecutive days. Doubling time was calculated using nonlinear regression of exponential (Malthusian) growth model. The results were average from two independent experiments. Statistical analysis was performed using multiple *t* test. Exact *p* values for the doubling time were depicted. AID, auxin-induced protein degradation; ChIP-seq, chromatin immunoprecipitation sequencing; EV, empty vector; IAA, indole-3-acetic acid; NELF, negative elongation factor.

Next, we used chromatin immunoprecipitation sequencing (ChIP-seq) to assess the impact of various NELFB MTs on Pol II pausing. First, we confirmed that chromatin binding of ectopic WT NELFB as detected by flag-based ChIP-seq was concordant with our previously published finding from ChIP-seq of endogenous NELFB (Figs. 2*A* and S3, *A* and *B*) (17). As expected, the flag-based ChIP-seq signal was largely eliminated in MT Δ-C60–expressing cells (Fig. 2, *A* and *C*), thus further validating the ChIP-seq specificity. Second, compared to WT cells, there was a substantial decrease in global Pol II density in all three MT MEFs examined (Figs. 2, *B* and *C* and S3, *C*–*E*). However, Pol II binding in Δ-C60 cells was reduced proportionally at both the transcription start sites (TSS) and GB (Fig. 2, *D* and *E*), resulting in negligible changes in the Pol II traveling ratio (TR) (Fig. S3*F*). In contrast, MT194 and MT486 MT cell clones displayed a preferential reduction of Pol II binding at TSS over GB (Fig. 2, *D* and *E*), giving rise to significantly diminished TRs (Fig. S3*F*). This suggests a multi-step nature of the NELF-dependent effects on Pol II movement. It is also worth noting that MT486, which lacks NELFE binding, was less defective in Pol II occupancy than MT194 (Fig. 2, *D* and *E*). Furthermore, unlike MT194, MT486 also retained significant chromatin binding at TSS (Fig. S3*G*), supporting the notion that disruption of the NELFB-E interaction is less consequential than that of NELFA-C-B. This is in line with the previous finding that the NELFE tentacle is not required to stabilize NELF complex at the TSS (41).

To rule out caveats associated with possible compensatory changes in stable MT cell clones, we established an auxin-induced protein degradation (AID) system in MEFs (Fig. S3*H*). Upon rapid depletion of endogenous WT NELFB, ectopic Δ-C60 failed to stabilize the other NELF subunits (Fig. 2*F*) or support their chromatin binding at TSS (Fig. S3, *I* and *J*). Likewise, unlike ectopic WT NELFB, Δ-C60 did not restore overall Pol II occupancy at either TSS or GB in NELFB-depleted cells (Fig. S2, *G*–*I*). Despite the total loss of its Pol II–pausing capability, Δ-C60 still supported cell proliferation as effectively as ectopic WT NELFB upon rapid depletion of endogenous NELFB (Fig. 2*J*). Taken together, these data clearly show that NELFB-dependent Pol II pausing and cell proliferation are functionally separable.

### NELFB-dependent transcriptome does not entirely depend on NELF chromatin binding or NELF-mediated Pol II pausing at TSS

**Figure 3:**
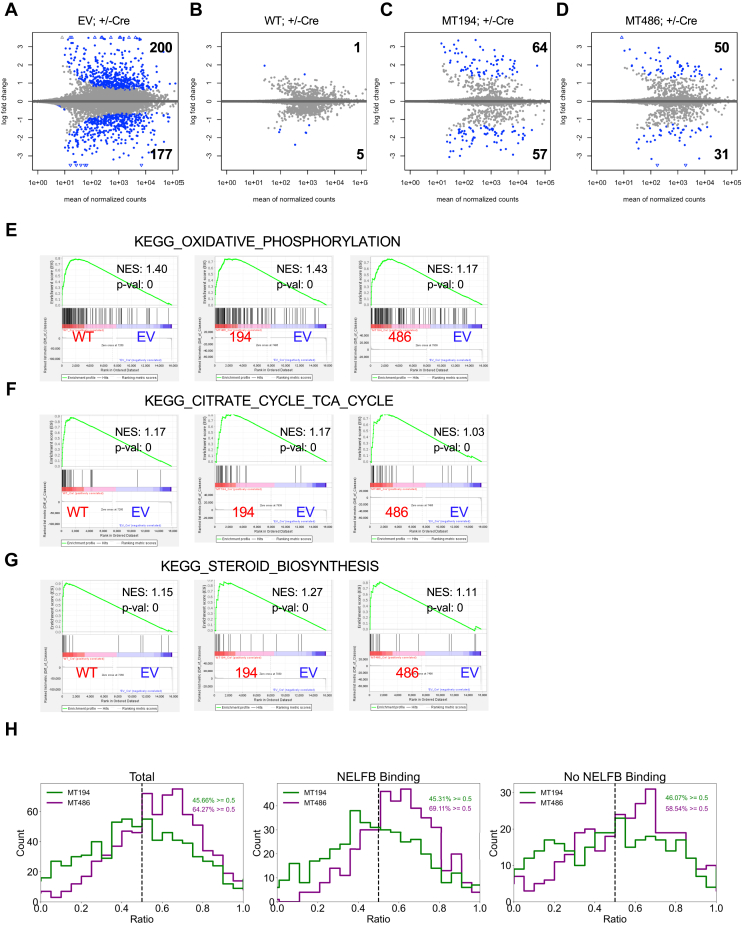
**NELFB-dependent transcriptome does not entirely depend on NELF chromatin binding or Pol II pausing at TSS.***A*–*D*, MA plots that compare RNA-seq of *Nelfb^f/-^* cells ± Cre treatment, with stable expression of EV (*A*), WT (*B*), MT194 (*C*), and MT486 (*D*). Genes with significant changes are indicated in *blue*, and the total gene numbers in both directions are shown for each pair of ± Cre conditions. RNA-seq was performed using two independent experiments. *E*–*G*, Gene Set Enrichment Analysis (GSEA) comparing ectopic WT, MT194, or MT486 with EV in NELFB-depleted cells. *H*, ability of the individual NELFB mutants to restore NELFB-dependent transcription of NELFB-bound (NELFB binding) and NELFB-unbound genes (no NELFB binding). For each individual gene whose transcription is dependent on NELFB, the restoration by EV and WT NELFB was set in *x*-axis as ratio 0 and 1, respectively. The counts of gene numbers in each interval are indicated as values on *y*-axis. Genes restored by MT194 and MT486 are indicated by *green and purple colors*, respectively. The percentages of genes that were restored over 50% (0.5) by either mutant were indicated in each graph. EV, empty vector; NELF, negative elongation factor.

Given the well-documented role of NELFB in transcriptional regulation, we asked to what extent the separation-of-function MTs could support NELFB-dependent transcriptome. Surprisingly, our initial transcriptomic study showed little difference between stable clones of WT NELFB *versus* Δ-C60–expressing MEFs (Fig. S3*K*), despite the lack of NELF chromatin binding and the compromised Pol II occupancy in Δ-C60–expressing KO cells (Fig. 2). To validate this finding with additional NELFB MTs and mitigate possible compensatory effects in stable clones, we performed RNA-seq using KO cell populations 7 days after Cre-mediated deletion of endogenous *Nelfb* (Fig. 1*D*). Consistent with previous studies (17, 18, 25), *Nelfb* KO cells displayed reduced expression of genes involved in metabolic pathways, including oxidative phosphorylation, the tricarboxylic cycle, and steroid biosynthesis (Fig. S4*A*). As expected, ectopic NELF WT in KO cells rescued expression of most KO-affected genes (compare Figs. 3, *A* and *B*, S4*B*, and Tables S2–S5, 98.4%). Despite their deficiency in binding to other NELF subunits and Pol II pausing, MT194 and MT486 MTs were capable of partially supporting transcription of NELFB-regulated genes (Figs. 3, *C* and *D*, S4*B* and Tables S2–S5, 67.9% and 78.5%, respectively). In further support, Gene Set Enrichment Analysis indicates that both ectopic WT and MT NELFB proteins are capable of supporting NELFB-upregulated and NELFB-downregulated pathways (Figs. 3, *E*–*G* and S4, *C* and *D*). Of note, neither MT194 nor MT486 MTs displayed any obvious preference in transcriptional rescue toward NELFB-bound genes in *Nelfb* KO MEFs (Fig. 3*H*), which is consistent with the lack of chromatin binding for MT194 (Fig. S3*F*). These data strongly suggest that NELFB-dependent transcriptome in MEFs does not entirely depend on the integrity of NELF complex, NELF chromatin binding, or Pol II pausing at TSS.

### NELFC binding–deficient MTs of NELFB are retained in the cytoplasm

**Figure 4:**
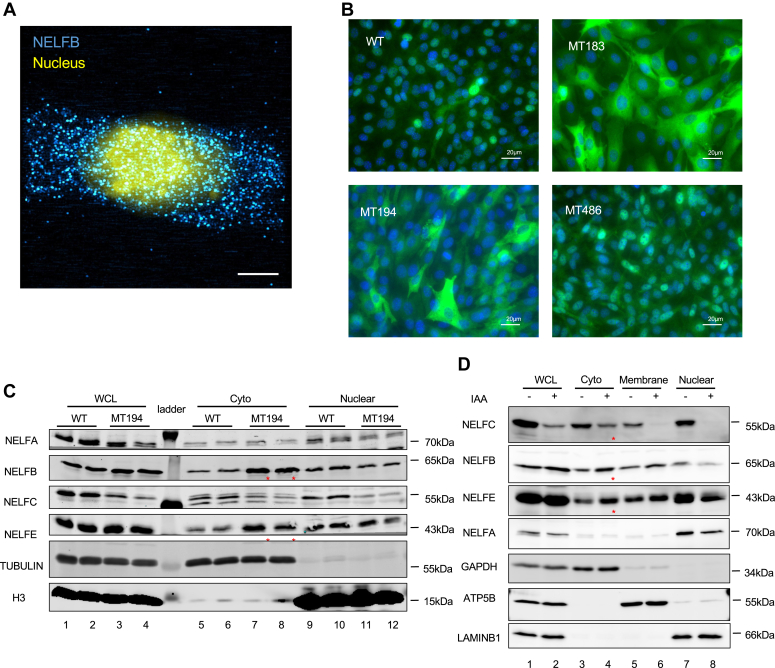
**NELFC binding-deficient mutants of NELFB are retained in the cytoplasm**. *A*, Super-resolution 3D structured illumination microscopy-based immunofluorescence image of NELFB (*blue*) and DAPI (*yellow*) in WT cells. Representative images from three independent repeats were shown. The scale bar represents 20 μm. *B*, immunofluorescent staining of NELFB and DAPI in WT, MT183, MT194, and MT486 cells. Representative images from three independent repeats were shown. The scale bar represents 20 μm. *C*, Western blotting of different NELF subunits in ectopic WT and MT194 clones. Tubulin and H3 are used as markers for cytosol and nuclear fractions, respectively. Two independent clones for each cell line were used and the experiments were done with two independent repeats. *Asterisks* indicate elevated levels of cytoplasmic NELFB and NELFE in MT194 cell clones. *D*, Western blotting of NELF subunits in NELFC-AID cells, following treatment of PBS or 0.5 mM IAA for 6 h. GAPDH, ATP5B, and LAMIN B1 were used as markers for cytosol, membrane, and nuclear fractions, respectively. Representative result is shown from three independent biological repeats. *Asterisks* indicate elevated levels of cytoplasmic NELFB and NELFE, despite the reduction of cytoplasmic NELFC. AID, auxin-induced protein degradation; DAPI, 4′,6-diamidino-2-phenylindole; IAA, indole-3-acetic acid; NELF, negative elongation factor.

Our data so far suggest a nongenomic function of NELFB. In support, super resolution 3D–structured illumination microscopy imaging showed that while WT NELFB was mainly present in the nucleus, a fraction of it was also detectable in the cytoplasm (Fig. 4*A* and Movie S1). By microscopy and/or biochemical fractionation, we also found that unlike WT, NELFB MTs defective in NELFC binding (MT183 and MT194) were mainly localized in the cytoplasm (Fig. 4, *B* and *C*). In contrast, MT486, which retained binding to NELFC and chromatin, was predominantly localized in the nucleus (Fig. 4*B*). Despite their subcellular mis-localization, the cytoplasmic MTs (MT183 and MT194) were competent in supporting cell proliferation (Figs. 1*E* and S5*A*). This is consistent with the notion that the Pol II pausing function of nuclear NELFB is not absolutely required for NELFB-dependent cell proliferation.

Compared to their WT counterparts, MT194-expressing MEFs displayed enriched cytoplasmic NELFE, but not NELFA or NELFC (Fig. 4*C*). Because MT194 lacks NELFC binding but still binds to NELFE, we hypothesized that NELFC could be responsible for nuclear retention of the NELFB-E subcomplex. To test this, we established a NELFC-AID cell line that can be induced for rapid NELFC degradation (Fig. S5*B*). Acute NELFC depletion led to accumulation of cytoplasmic NELFB and NELFE and concurrent reduction of their nuclear pools (Fig. 4*D*). The effect of NELFC depletion on stabilizing NELFB persisted 3 days after incubation with auxin (Fig. S5*C*). These data strongly suggest that NELFC is required for nuclear recruitment and/or retention of NELFB-E. However, cytoplasmic NELFB is likely sufficient to support cell proliferation.

### NELFB is physically and functionally associated with signaling molecules

**Figure 5:**
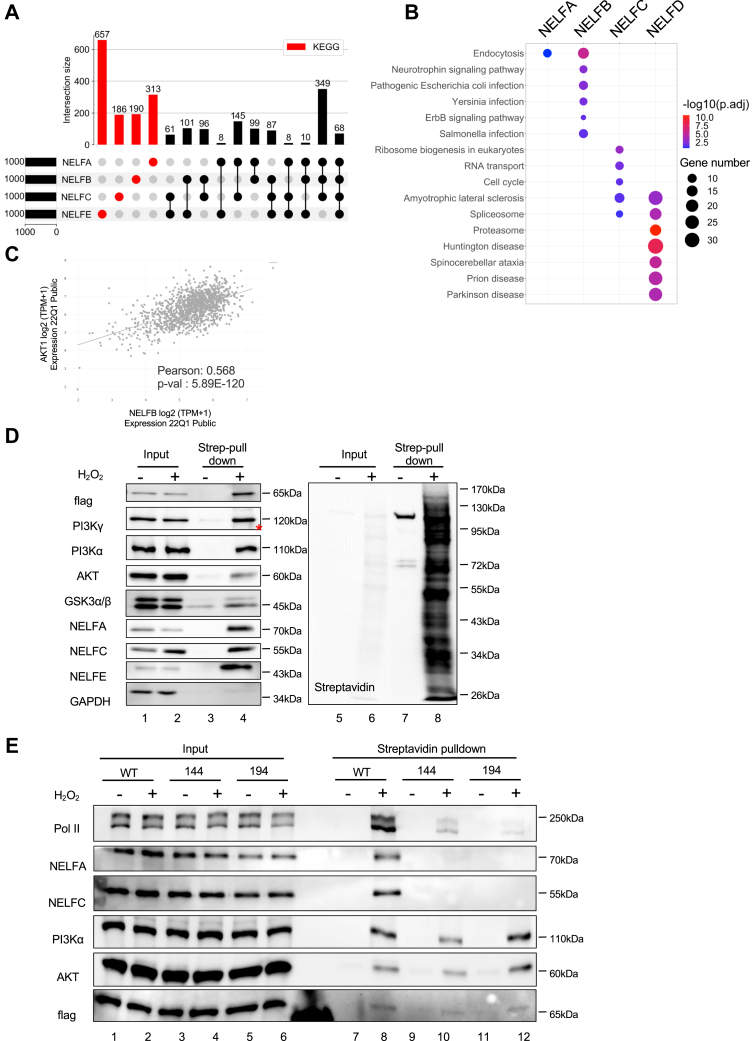
**NELFB is physically associated with signaling molecules.***A*, *UpSet plot* showing the overlapping coexpressing genes for individual NELF subunits. The top 1000 coexpressing genes were generated from DepMap Project Expression 22Q1 Data including 1393 cell lines. The *red color* highlights groups of nonoverlapping coexpressing genes that are unique to each NELF subunit. *B*, Kyoto Encyclopedia of Genes and Genomes (KEGG) pathway enrichment for nonoverlapping coexpressing genes that are unique to each NELF subunit in (*A*). The number of enriched genes is shown with different sizes and the *p*-value of the enrichment indicated with different colors. *C*, *correlation plot* presenting the relationship between AKT1 and NELFB expression for each cell line in DepMap Data Expression 22Q1 Public (a total of 1393 cell lines). The regression line with Pearson of 0.568 is shown. *D*, a stable cell line with C-terminal APEX2-tagged NELFB was used for proximity labeling. Input and pull-down lysate by streptavidin conjugated beads were analyzed by Western blotting for signaling molecules and NELF subunits. The blot was also probed with streptavidin to show the overall enrichment of biotinylated protein. Control cells were not treated with 1 mM H_2_O_2_. Representative results are shown from three independent repeats. The band of pull-down lane in PI3Kγ plot is indicated by an *asterisk*. *E*, stable NELFB–AID cell lines with C-terminal APEX2-tagged WT, MT144, or MT194 NELFB were used for proximity labeling. NELFB-AID protein was degraded by IAA for 2 h before the labeling experiment. Input and pull-down lysate by streptavidin conjugated beads were analyzed by Western blotting. Representative results come from three independent repeats. AID, auxin-induced protein degradation; APEX2, apurinic/apyrimidinic endodeoxyribonuclease 2; IAA, indole-3-acetic acid; NELF, negative elongation factor; PI3K, phosphatidylinositol-3-kinase.

Recent work demonstrates that co-expression of genes is informative for functional interactions (42). The top 1000 genes that display a positive correlation in expression with NELFB are enriched with players in RNA metabolism–related pathways, including mRNA splicing and RNA transport (Table S6). In addition, significantly enriched genes also include those involved in several important signaling pathways such as neurotrophin, AMP-activated protein kinase, T cell recepto, insulin, ErbB, sphingolipid, tumor necrosis factor, hypoxia-inducible factor-1, and mammalian target of rapamycin signaling pathways (Table S6). Because our data suggest that the progrowth function of NELFB is independent of the NELF complex, we focus on the 190 genes that exhibit positive correlation uniquely with NELFB, but not the other NELF subunits (Fig. 5*A*). These NELFB-specific co-expressing genes are enriched in endocytosis, neurotrophin, and ErbB signaling pathways (Fig. 5*B* and Table S7). For example, NELFB expression is positively correlated with a few of prosurvival signaling molecules, including PDPK1, AKT1/2, mammalian target of rapamycin, RPTOR, TSC1 in the PI3K/PDK1/AKT/mammalian target of rapamycin pathway, and RAF1, MAP2K7, MAPK1 in the RAF/MEK/MAPK pathway (Figs. 5*C* and S6, *A*–*C*). Based on a functional prediction method from GeneMANIA (43), these molecules are predicted to have physical and/or genetic interactions among themselves (Fig. S6*D*). Using apurinic/apyrimidinic endodeoxyribonuclease 2 (APEX2)-mediated proximity labeling (44), we detected proximal association of NELFB with a few of the signaling molecules *in situ*, including PI3Kα and AKT (Figs. 5*D* and S7*A*). Consistent with our co-IP result, NELF MTs defective in NELFC binding and mis-localized in the cytoplasm (MT144 and MT194) did not exhibit proximity with NELFA, NELFC, or Pol II (Fig. 5*E*). However, the same MTs retained the physical proximity with PI3Kα and AKT (Fig. 5*E*). Thus, our results strongly suggest that NELFB’s association with signaling molecules can be uncoupled from its interactions with its known nuclear partners involved in Pol II pausing.

**Figure 6:**
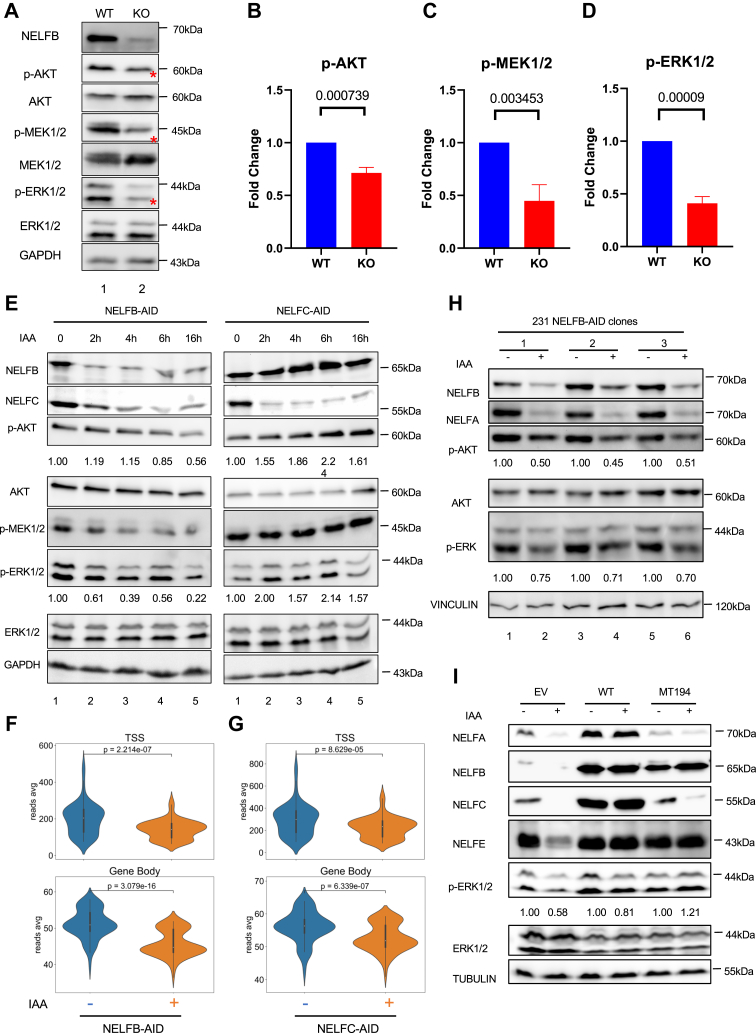
**NELFB supports progrowth signaling transduction.***A*, Western blotting of cell lysates from MEFs depleted of NELFB by Cre-loxP. Representative results from three independent repeats. *Asterisks* indicate the reduced intensity of phosphorylated proteins. *B*–*D*, quantification of phosphorylation of various signaling molecules, which was normalized with total proteins from three independent experiments. Statistics was conducted using multiple *t* test. *E*, NELFB-AID and NELFC-AID MEFs were treated with PBS or 0.5 mM IAA and then harvested at different time points. Cell lysates were analyzed by Western blotting. The intensity of phosphorylation was normalized with total protein and indicated below the corresponding protein. Representative images were shown from three independent repeats. *F* and *G*, *violin plot distributions* of Pol II ChIP-seq reads at the TSS (−0.5 to +0.5kb) and GB (+0.5 to +2.5 kb) in NELFB-AID cells (*F*) and NELFC-AID cells (*G*) following treatment of PBS or 0.5 mM IAA for 6 h. Pol II ChIP-seq was from two independent experiments. *H*, NELFB-AID in MDA-MB-231 cells following IAA treatment. Cell lysates were analyzed by Western blotting. The intensity of phosphorylation was normalized with total protein Three independent clones were used for the experiment. *I*, NELFB-AID cell lines that stably expressed EV, WT, or MT194 were treated with IAA. Cell lysates were analyzed by Western blotting. The intensity of phosphorylation was normalized with total protein. Representative images from three independent repeats are shown. AID, auxin-induced protein degradation; ChIP-seq, chromatin immunoprecipitation sequencing; EV, empty vector; IAA, indole-3-acetic acid; MEF, mouse embryonic fibroblast; NELF, negative elongation factor.

To determine whether NELFB has any influence on the activity of these signaling molecules, we compared the phosphorylation status of several key signaling molecules in *Nelfb* WT and Cre/loxP-induced KO cells. There was a significant reduction in p-AKT, p-MEK1/2, and p-ERK1/2 in KO cells *versus* WT (Fig. 6, *A*–*D*). Notably, NELFB depletion did not affect either mRNA or total protein levels of these signaling molecules (Figs. 6*A* and S7*B*), thus ruling out a direct role of NELFB in transcriptional regulation of these genes. Using the AID-based rapid degradation system, we found that reduction in phosphorylation of these signaling proteins occurred as early as 2 h after NELFB depletion (lanes 1–5, Fig. 6*E*). In contrast, rapid NELFC depletion resulted in increases in the phosphorylation intensity (lane 6–10, Fig. 6*E*), likely due to increased cytoplasmic retention of NELFB (Fig. 4*D*). Despite their differential effects on the signaling molecules, both NELFB and NELFC depletion showed similar reduction of Pol II occupancy at TSS and GB (Figs. 6, *F* and *G* and S7*C*). A similar effect of rapid NELFB degradation on p-AKT was observed in human cells (Figs. 6*H* and S1*D*). Lastly, we found that both WT NELFB and MT194, a MT predominantly retained in the cytoplasm, were capable of rescuing phosphorylation of these signaling molecules in *Nelfb* KO MEFs (Fig. 6*I*). Thus, our data strongly suggest physical and functional interactions between cytoplasmic NELFB and several progrowth signaling kinases, which can be genetically separable from the NELF nuclear function.

### Enforced activation of the PI3K/AKT pathway rescued the proliferation defects of NELF-depleted MEFs

**Figure 7:**
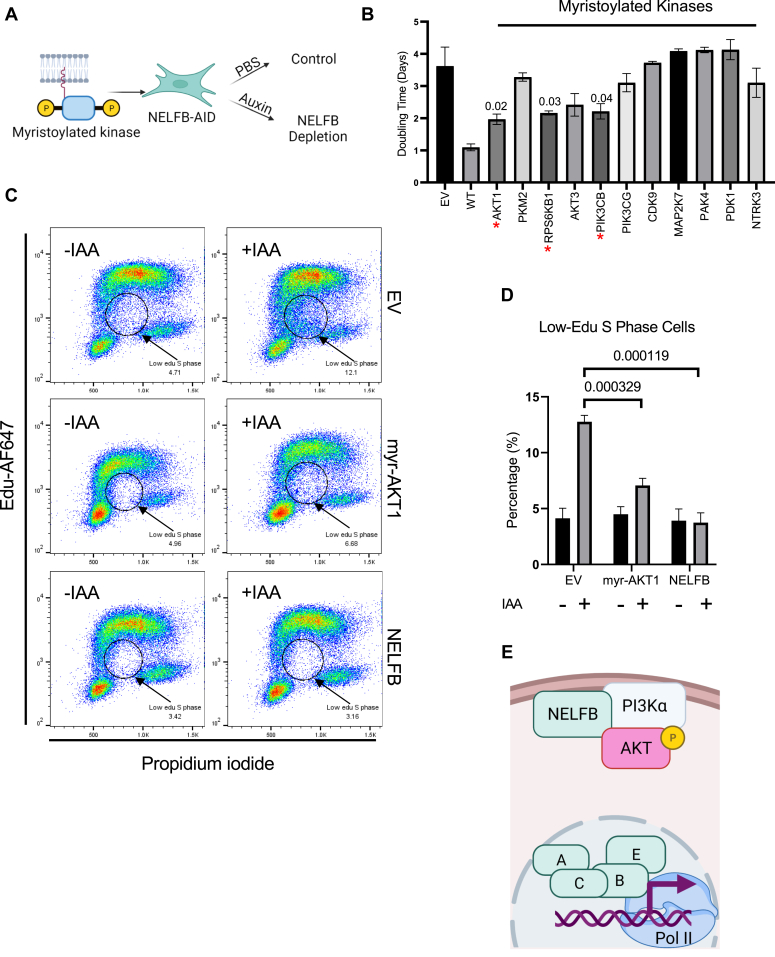
**Enforced activation of the PI3K/AKT pathway rescued the growth defects of NELF-depleted MEFs.***A*, schematic illustration for the myristoylated (Myr) kinase screen. *B*, NELFB-AID cell lines with ectopic Myr kinases were treated with IAA for four consecutive days. Cell growth was measured. Doubling time was calculated using nonlinear regression of exponential (Malthusian) growth model. Statistical analysis was performed using multiple *t* test to compare kinase group with EV, and *p*-value less than 0.05 was indicated. The results were average from two independent biological repeats. *C*, flow cytometry of cells from the click-it experiment, stained for EdU-AF647 and propidium iodide (PI). Stable NELFB–AID cells expressing EV, Myr-AKT1, or WT NELFB were treated with IAA before the pulse experiment. The S phase populations with low Edu are highlighted and the percentages are shown. Representative results from three independent experiments are shown. *D*, average quantification of the low-Edu S phase cells from three independent experiment from (*D*). Statistical analysis was performed using multiple *t* test to compare IAA treated myr-AKT1 or NELFB with EV groups. The *p*-value is indicated. *E*, diagram shows the molecular interaction between NELFB and PI3K/AKT pathway. AID, auxin-induced protein degradation; EdU, 5-ethynyl-2′-deoxyuridine; EV, empty vector; IAA, indole-3-acetic acid; MEF, mouse embryonic fibroblast; NELF, negative elongation factor; PI3K, phosphatidylinositol-3-kinase.

Myristoylation-mediated membrane recruitment is known to enforce activation of various cytoplasmic kinases including PI3Kα and AKT (45, 46). We therefore surmised that ectopic expression of membrane–tethered, NELFB-interacting kinases could mitigate the proliferative deficiency of NELF-depleted cells. To test our hypothesis, we introduced a few of myristoylated (Myr) kinases in the PI3K/AKT pathway (AKT1, RPS6KB1, AKT3, PIK3CB, and PIK3CG) into NELFB-depleted cells (Fig. 7*A*) (47). In addition, several previously reported NELFB-associated kinases were also included in our experiment (CDK9, MAP2K7, PAK4, PKM2, and NTRK3) (18, 30, 48). In support of our hypothesis, Myr-AKT1, RPS6KB1, and PIK3CB partially restored the defects in cell proliferation upon NELFB depletion (Fig. 7*B*).

Ectopic expression of Myr-AKT1 in NELFB-depleted MEFs did not increase the abundance of NELFB or the other three NELF subunits (Fig. S8*A*). Therefore, the rescue of cell proliferation by Myr-AKT1 was unlikely due to augmentation of NELF expression. It is also noteworthy that although Myr-AKT1 fully restored phosphorylation of GSK3β, a direct downstream target of AKT, phosphorylation of MEK1/2 and ERK1/2 was still compromised in NELFB-depleted cells with Myr-AKT1 overexpression (Fig. S8*A*). Furthermore, Pol II ChIP-seq showed that Myr-AKT1 overexpression in NELFB-depleted cells did not restore the loss of Pol II occupancy at either TSS or GB (Fig. S8, *B* and *C*). Thus, the rescuing effect of Myr-AKT1 on cell proliferation was not due to restoration of NELF-dependent Pol II pausing.

We previously reported that NELFB-depleted cells exhibited defects in cell cycle progression in multiple phases including S-phase DNA replication (17). Consistent with our published finding, 5-ethynyl-2′-deoxyuridine (EdU) pulse labeling showed an increased population of low EdU–labeled S-phase cells upon NELFB depletion (Fig. 7, *C* and *D*). Ectopic expression of either WT NELFB or Myr-AKT1 substantially reduced this population of cells (Fig. 7, *C* and *D*), suggesting that AKT1 acts downstream of NELFB to promote cell cycle progression. Taken together, our results support the notion that the function of NELFB in supporting cell proliferation is at least partially mediated by key signaling molecules in the PI3K/AKT pathway.

## Discussion

Biochemical and genetic approaches are complementary to each other in elucidating the functional relationships in biological processes (49). A wealth of biochemical evidence establishes a central role of the NELF complex in mediating genome-wide Pol II pausing during transcription elongation (1, 50). On the other hand, genetic studies from our group and others clearly demonstrate vital roles of individual NELF subunits in development, differentiation, and homeostasis in a variety of tissues (17, 18, 19, 20, 21, 23, 24, 25, 26, 27, 28, 51). Thus, it has been a prevailing presumption that NELF’s Pol II pausing activity underlies its essentiality in biological functions, including cell proliferation. Using a panel of separation-of-function MTs and a rapid protein degradation approach, our current work shows that neither the integrity of the NELF complex nor its Pol II pausing activity is essential for NELFB-dependent cell proliferation or the bulk of its transcriptome. More specifically, these NELFB MTs can largely substitute WT NELFB in supporting cell proliferation and NELF-dependent transcription, despite their defects in assembly of an intact NELF complex and control of promoter-proximal Pol II pausing. This separation-of-function phenotype is perhaps most pronounced for MT194 and Δ-C60. The former lacks NELFC binding and is predominantly localized in the cytoplasm, whereas the latter does not bind to NELFE and is expressed at a barely detectable level. Not surprisingly, neither MT binds to TSS nor effectively pauses Pol II in MEFs. Nevertheless, they largely rescue the proliferative and transcriptional defects in *Nelfb* KO cells. Therefore, by genetically uncoupling NELFB’s function in cell proliferation from its role as part of an intact NELF complex in Pol II pausing, our findings highlight the multifaceted nature of NELFB and thus call for the need to reevaluate the preponderant Pol II pausing–centric paradigm in NELF biology.

Based on the ortholog analysis, the four NELF subunits do not appear to have evolved simultaneously (Fig. S8*D*) (52). For example, orthologs of both NELFB and NELFC, but not those of NELFA and NELFE, are found in multiple single-cell organisms including *Fungi*, *Protista*, and *Viridiplantae*, in which pausing has not been documented (53). Thus, NELFB and NELFC are likely endowed with functions independent of the other two NELF subunits. Furthermore, *Hydra*, an early metazoan, expresses the orthologs of all four NELF subunits yet exhibits no apparent genome-wide Pol II pausing at TSS (54). Taken together, these observations are in line with the notion that NELFB orthologs in these lower organisms may possess biological properties more primal than NELF complex–dependent Pol II pausing.

Our data strongly indicate that cytoplasmic NELFB supports cell proliferation by physically associating with and activating progrowth signaling molecules such as PI3K/AKT. This is distinct from the previously reported role of NELF in transcription of the genes involved in the MAPK pathway (18) for the following reasons. First, NELFB MTs MT144 and MT194, which are defective in associating with NELFA, NELFC, and Pol II, still maintain their physical interactions with PI3Kα and AKT. Second, rapid degradation of NELFB, but not NELFC, attenuates PI3K-AKT and MEK-MAPK activities without affecting the protein or mRNA abundance of these kinases. Third, membrane tethering of PI3K-AKT kinases partially rescues the defects of cell proliferation without enhancing Pol II occupancy in NELFB-depleted cells, providing further evidence for two distinct NELFB functions. NELFB likely targets additional kinases besides AKT, which could account for the partial restoration of the proliferative defects by enforced AKT activation. In support of this possibility, Myr-AKT1 did not restore pMEK1/2 or pERK1/2 in *Nelfb* KO cells (Fig. S8*A*). This nongenomic activity of NELFB, which could be evolutionarily more conserved than its Pol II pausing activity, may also contribute to various context-dependent functions of NELFB in multicellular organisms *in vivo*.

Paused Pol II is considered as an equilibrium between Pol II recruitment, early termination, and release (4). NELF, together with the Integrator complex and cap-binding complex, has been shown to coordinate Pol II pausing, mRNA modification, and premature termination (11, 15, 55, 56). In this regard, it is worth noting that different NELFB MTs as characterized in our study exhibit distinct effects on the equilibrium of Pol II pausing. For example, Δ-C60 displays defects of Pol II occupancy at both TSS and GB, which could imply a defect in facilitating promoter recruitment of Pol II. In contrast, MT486, which is defective in binding to NELFE, reduces Pol II chromatin occupancy only at TSS, but not GB. This indicates that a partially assembled NELF complex, albeit incapable of pausing Pol II at TSS, may still recruit sufficient Pol II and release it to GB.

Our findings revise, but do not negate, the current understanding of the physiological significance of Pol II pausing. It will be of interest to determine the relative contributions of the genomic and nongenomic activities of NELFB to NELFB-dependent regulation of various cellular processes (Fig. S8*E*). NELF subunits are known to undergo dynamic shuttling among different subcellular compartments (31, 57). In particular, we surmise that NELFB translocation from the nucleus to cytoplasm could be driven by the conserved nuclear export sequences (Fig. S8*E*) and a yeast exportin1–like domain in NELFB, following its dissociation from NELFC (13, 58). We further propose that dynamic regulation of the relative abundance of the nuclear and cytoplasmic NELFB could coordinate the actions of these two functionally distinct NELFB pools.

Pol II pausing has been implicated in coordinating gene activity in both spatial and temporal manners, especially transcriptional responses to developmental and environmental stimuli (1, 50). While our work clearly indicates that Pol II pausing is not entirely responsible for NELFB-dependent transcriptome in cultured MEFs under regular condition, it will be of importance to examine the transcriptomic effects of the separation-of-function NELFB MTs in other physiological contexts.

In summary, our work demonstrates a previous unrecognized progrowth function of cytoplasmic NELFB, which is clearly independent of an intact NELF complex and its cardinal role in Pol II pausing. Compared to the gene KO approach, separation-of-function MTs offer a clear advantage in uncoupling the nongenomic *versus* genomic functions of NELFB. Further work is warranted to further elucidate the biochemical and structural basis for NELFB-mediated regulation of the progrowth signaling molecules, which could in turn inform novel therapeutic targets for anticancer treatment.

## Experimental procedures

### Plasmid construction

Cloning was performed using In-Fusion kit (Takara, 638,947). C-terminal 3× flag-tagged mouse NELFB was introduced into the pLenti-CMV-GFP-neo (Addgene: 17447) backbone. NELFB truncations and site-specific point mutagenesis were generated using PrimeSTAR GXL DNA Polymerase (Takara, R050B) and different sets of primers (Tables S1 and S10). OsTIR1 was cloned into pCDH-CMV-mcherry-T2A-puro (Addgene: 72264). C-terminal mini-AID–tagged mouse NELFB and human NELFC were cloned into pLenti-6.3/V5-DEST-GFP (Addgene: 40125). C-terminal AID–tagged human NELFB with silent mutation resistant to single guide RNA (sgRNA) was cloned into pLenti-6.3/V5-DEST-GFP. APEX2 was cloned to the C-terminal of WT and MTs (MT144 and MT194) NELFB into pLenti-CMV-GFP-neo backbone (Table S8). Sequences of all constructs were verified by sequencing.

### Cell culture

Immortalized *Nelfb*^*flox/-*^ mouse embryonic fibroblasts (iMEFs) were described previously (17). MEFs, MDA-MB-231, and Lenti-X 293T (Takara: #632180) cells were cultured in Dulbecco's modified Eagle's medium supplemented with 10% fetal bovine serum (Gibco: 10099141), 100 U/ml penicillin, and 100 mg/ml streptomycin (Gibco: 10,378,016). All cells were grown in a 37 °C humid incubator with 5% CO2. *Mycoplasma* detection was carried out on a regular basis.

### Stable cell lines establishment and colony isolation

*Nelfb*^*flox/-*^ iMEFs were used to generate the following stable cell lines and to isolate single colonies. First, an empty vector or C-terminal 3×flag-tagged NELFB (WT, Δ-C60, Δ-N78, MT144, MT183, MT194, and MT486) was introduced into *Nelfb*^*flox/-*^ iMEFs by lentiviral infection and selected with 800ug/ml G418 (Gibco: 10131027). The established cell lines were used in the Cre-induced NELFB MT-swapping experiments. After removing the endogenous floxed *Nelfb* allele by Adeno-Cre, single colonies were isolated and propagated from WT and MT stable cell lines.

To establish the NELFB-AID system in MEFs, NELFB with C-terminal mini-AID-tag (pLenti-mNELFB-AID-BSD) and OsTIR1-mcherry (pCDH-OsTIR1-mcherry-T2A-puro) were cointroduced into *Nelfb*^*flox/-*^ iMEFs *via* lentiviral infection and selected in media containing 5ug/ml Blasticidin (Gibco: A1113903) and 2ug/ml puromycin (Gibco: A1113803). Single colonies were isolated upon deletion of the endogenous floxed *Nelfb* allele by Adeno-Cre. To establish NELFB MT stable cell lines on top of NELFB-AID parental cells, cells were further infected with lentiviruses expressing NELFB, APEX2-NELFB, or the following MTs: Δ-N78, Δ-C60, MT144, MT183, MT194, APEX2-MT144, or APEX2-MT194. To establish Myr Kinases stable cell lines on top of the NELFB-AID parental cells, cells were infected with retroviruses expressing the following kinases: AKT1, PKM2, RPS6KB1, AKT3, PIK3CB, PIK3CG, CDK9, MAP2K7, PAK4, PDK1, and NTRK3 (Addgene, 1000000012).

For NELFC-AID cells, human NELFC with the mini-AID tags (pLenti-HNELFC-AID-BSD) was introduced into NELFB-WT iMEFs or NELFB-Δ-C60 iMEFs, together with OsTIR1-mcherry (pCDH-OsTIR1-mcherry-2A-puro). Single colonies were isolated upon deletion of endogenous NELFC gene by Cas9 and sgRNA transfection (Table S9).

The following cell lines and single colonies were established in human MDA-MB-231 breast cancer cell line. To establish NELFB-Δ-C60 MDA-MB-231 cells, sgRNA targeting the C terminus of NELFB was cotransfected with Cas9 into parental MDA-MB-231 cells. Single colonies were isolated and screened for the correct gene editing. To establish NELFB-AID MDA-MB-231 cells, silent MT NELFB-AID resistant to sgRNA targeting WT NELFB (pLenti-hNELFB-SM-AID-BSD) was first introduced into OsTIR1 stable MDA-MB-231 cells. Single colonies were then isolated upon deletion of the endogenous *NELFB* gene by Cas9 and sgRNA transfection (Table S9).

### Lentiviral, retroviral, and adenoviral infection

The lentiviral infection procedure was described previously (17). Briefly, 2.5 ug lentiviral plasmids were transfected into four million Lenti-X293T cells with 1 ug pMD2.G (Addgene, 12259) and 2ug psPAX2 (Addgene, 12260) using Lipofectamine 2000 (Invitrogen, 11668030). Supernatants were harvested and used to infect target cells with 8 ug/ml polybrene (Sigma, TR-1003) for overnight incubation at 37 °C. The procedure was the same for retroviral infection, except that 1 ug VSV.G (Addgene, 14888) and 2 ug gag/pol (Addgene, 14887) were used as packing plasmids instead. Retroviral infection was performed using a plate centrifuge at 1,500*g* for 4 h with 8 ug/ml polybrene. For adenoviral transduction, 1.5 × 105/well cells were seeded in a 6-well plate and adenovirus (UI Viral Vector Core Facility: VVC-U of Iowa-1174) was added at 100 pfu/cell in the media for overnight at 37 °C.

### Cell proliferation assays

MEFs or MDA-MB-231 cells were seeded in 96-well plates at a concentration of 1500 cells/well. Auxin, if indicated, was added at a final concentration of 0.5 mM after cells were attached to the plate and incubated for a period of time as indicated. Cell Counting Kit-8 (Dojindo: CK04) was diluted 1:10 with 10% fetal bovine serum Dulbecco's modified Eagle's medium media and incubated with cells for 1 h. *A*_450_ reading was taken using BioTek Synergy H1(BioTek, 13629) every 24 h for 4 days. *A*_450_ readings were processed using GraphPad Prism https://www.graphpad.com/. Doubling time was generated using nonlinear regression of Exponential (Malthusian) growth.

### Antibodies

The following antibodies were used in our study. NELFB (Cell Signaling, 14894), NELFB (Proteintech, 16418-1-AP), NELFC (Cell Signaling, 12265), NELFE (Proteintech, 10705-1-AP), NELFA (Proteintech, 10456-1-AP), flag (Sigma, F3165), Tubulin (Millipore, 05-829), PI3 Kinase p110α (C73F8) (Cell Signaling, 4249), PI3 Kinase p110γ (D55D5) (Cell Signaling, 5405), Akt (pan) (C67E7) (Cell Signaling, 4691), p-Akt (Ser473) (D9E) XP (Cell Signaling, 4060), p-GSK-3β (Ser9) (D85E12) (Cell Signaling, 5558), GSK-3α/β (D75D3) (Cell Signaling, 5676), p-MEK1/2 (Ser217/221) (Cell Signaling, 9121), ERK1/2 (Cell Signaling, 4695), p-ERK1/2(Thr202/Tyr204) (Cell Signaling, 4370), ATP5B(Millipore, MABS1304), LaminB1(Santa Cruz, sc-374015), GAPDH (Bio-Rad, 12004167), ACTIN (Bio-Rad, 12004163), Vinculin (Proteintech, 66305), and Streptavidin-IRDye 800CW (Licor, 926-32230). Secondary antibodies include IRDye 680RD Goat anti-rabbit immunoglobulin G (Licor, 926-68071), IRDye 800CW Goat anti-mouse immunoglobulin G (Licor, 926-32210), and Goat anti-rabbit immunoglobulin G (H + L)-horseradish peroxidase (Invitrogen, 31460).

### Western blotting and subcellular fractionation

Protein samples were prepared using Laemmli buffer with protease/phosphatase inhibitors (Cell Signaling, 5872). After lysis, protein concentration was quantified using a bicinchoninic acid protein assay kit (Pierce, 23225). SDS-PAGE and Western blotting procedure were described previously (25). Western blots were imaged using either Licor Odyssey (Licor, 9120) or ChemiDoc MP (Bio-Rad, 12003154).

Subcellular fractionation was performed using Cell Fractionation Kit (Cell Signaling, 9038) following the manufacturer’s instructions. GAPDH, ATP5B, and LaminB1 were used as cytoplasm, membrane, and nuclear markers, respectively.

### Coimmunoprecipitation

Whole-cell lysate was obtained by lysing cells in 500 ul NP-40 lysis buffer (50 mM Tris–HCl, pH 8, 4 mM EDTA pH 8, 150 mM NaCl, 1% NP40) with a protease/phosphatase inhibitor cocktail (Cell Signaling, 5872). Lysate was kept on ice for 15 min. Insoluble material was removed by centrifugation at 10,000*g* for 10 min. Lysates were then incubated with flag M2 magnetic agarose beads (15 ul/rxn, Sigma, M8823) at 4 °C overnight. Immune complexes were collected using a magnetic separator and washed three times with lysis buffer. To elute immunoprecipitated proteins, 3× flag peptide (Sigma, F4799) was diluted at a final concentration of 200 ng/μl in Tris-buffered saline (50 mM Tris–HCl pH7.4, 150 mM NaCl) buffer and incubated with beads for 30 min at 4 °C. The eluted lysates were analyzed by SDS-PAGE and Western blots as described.

### RNA isolation, RNA-seq, and realtime RT-quantitative polymerase chain reaction analysis

Total RNA isolation was performed using a RNeasy Plus Mini kit (Qiagen, 74034). For mRNA-Seq (with PolyA selection), libraries were constructed and sequenced at the University of Texas Health San Antonio Genome Sequencing Facility using 50 bp single-read sequencing. For RT-quantitative polymerase chain reaction (qPCR), RNA was reverse-transcribed into complementary DNA using a Maxima H Minus First Strand cDNA Synthesis kit (Invitrogen, A48571). Each cDNA sample was amplified using Thermo Fisher Scientific Luminaris Color HiGreen qPCR Master Mix, high ROX (Invitrogen, FERK0364) in CFX384 Touch Real-Time PCR Detection System (Bio-Rad, 1855484) following the manufacturer’s instructions and using specific primers (Table S4). Relative RNA levels were calculated using the 2−ΔΔCt method with the Ct values normalized using 18sRNA as an internal control.

### Immunofluorescent staining and super resolution imaging

WT, MT183, MT194, and MT486 MEFs in *Nelf-b* null background were generated as described above. 1.5 × 10^5^ cells were seeded in a glass-bottom dish 35 mm (Ibidi, 81218-200) for overnight at 37 °C. Cells were then fixed using 4% paraformaldehyde/PBS at room temperature (RT) for 15 min and permeabilized with 0.5% Triton X-100/PBS for 10 min. Permeabilized cells were then blocked in blocking buffer (PBS with 10% goat serum) for 1 h at RT and incubated with 1:50 NELFB antibody (Cell Signaling, 14894) at 4 °C overnight in blocking buffer. Cells were washed three times, 10 min for each with PBS, and incubated with an Alexa 568–conjugated anti-rabbit secondary antibody (Invitrogen, A11036; 1:1000 dilution) in the blocking buffer for 1 h. Cells were washed three times for 10 min in PBS before stained with 4′,6-diamidino-2-phenylindole (DAPI) 0.1 μg/ml in PBS for 10 min. DAPI was then removed and cells were imaged using Nikon Eclipse Ni fluorescent microscope.

For confocal imaging of NELFB distribution relative to nuclei, a Yokogawa CSU-X1 Spinning Disk Unit (Andor Technology) with an iXon DU-897-BV monochrome CCD (Andor Technology) was used. The imaging environment was maintained at 5% CO_2_, 37  °C. Image rendering was performed using ImageJ (https://imagej.net/ij/) (NIH). The 3D-structured illumination microscopy microscope was custom-built by the Chung Lab on a Zeiss Axio Observer inverted microscope platform with an ASI motorized stage and a 100×/1.46 NA oil immersion Zeiss objective (Alpha Plan-APO). NELFB excitation was performed at 568 nm and 405 nm excitation for DAPI, respectively. Sequential excitation at the two wavelengths was enabled by a software-controlled filter wheel (Finger Lakes Instrumentation). Excitation grating patterns at each wavelength were generated by a spatial light modulator (Forth Dimension Display) as previously described (59). Fluorescence images were collected using an sCMOS camera (Hamamatsu Flash 4.0) with an exposure time of 30 ms. 3D reconstruction of the raw data was performed using a custom software as previously described (59, 60).

### ChIP, library preparation, and qPCR

For ChIP-seq assay, cells were treated with indole-3-acetic acid at a final concentration of 0.5 mM for 6 h, if indicated. Then, cells were crosslinked with 1% formaldehyde/PBS for 10 min at RT and then quenched by adding glycine to a final concentration of 125 mM for 5 min at RT. After washed with cold PBS three times, cells were then resuspended in PBS at 50 million/ml. Same volume of 2× lysis buffer (100 mM Tris–HCl, pH 7.5, 100 mM NaCl, 30 mM MgCl2, 4% Triton X-100) with protease inhibitors (1 μg/ml leupeptin, 1 μg/ml aprotinin, and 1 μg/ml pepstatin) was added to the cell suspension and incubated on ice for 10 min. Chromosomal DNA was fragmented with micrococcal nuclease (NEB, M0247S) at 50 unit/ul and incubated at 37 °C for 15 min. The reaction was stop with EDTA and EGTA at 5 mM each and sonicated using Qsonica Sonicator Q500 for 40 s with 0.5 S on, 0.5 S off at 40%. Supernatant was collected by centrifuging at 14,000*g* for 10 min then diluted with ChIP-lysis buffer (50 mM Tris–HCl, pH 8.0, NaCl 150 mM, 2 mM EDTA) with protease inhibitors. Antibodies used for ChIP include anti-Pol II (2 ug/rxn, BioLegend; 664906) and anti-flag (2 ug/rxn, Sigma, F3165), NELFC (5 ul/rxn Cell Signaling, 12265), NELFE (5 ul/rxn, Abcam, ab170104). Diluted chromatin was incubated with antibodies at 4 °C overnight. Dynabeads Protein A and Protein G (Invitrogen, 10015D) was added the following day and incubated for 2 h at 4 °C. After incubation, Dynabeads was washed as previously described (51). Samples were subsequently eluted and reverse-crosslinked at 65 °C overnight in Elution Buffer (50 mM Tris–HCl pH8.0, 1% SDS, 10 mM EDTA pH 8.0). ChIP DNA was recovered as described (25) and library was built using Kapa Hyperprep Kit according to the instructions (Roche, KK8502). Library quality was confirmed by high-sensitivity DNA ChIP with Bioanalyzer (Agilent, 5067-4626), to ensure that samples had a single peak around 380 bp. Libraries were then quantified by Qubit and pooled for 50 bp single-end sequencing (Illumina HiSeq 3000). Library sequencing was conducted at University of Texas Health San Antonio Genome Sequencing Facility.

### Proximity labeling by APEX2

Proximity labeling by APEX2 was carried out following a published protocol (44). Five million cells per sample were seeded in a 15 cm plate. After 48 h, auxin was added at 0.5 mM, and cells were incubated for 4 h to deplete AID-tagged NELFB protein before labeling. 2.5 mM biotin-phenol in dimethylsulfoxide was added to the media for 30 min at 37 °C. Cells were then washed with PBS (with 1 mM CaCl_2_, 0.5 mM MgCl_2_) three times and then incubated with or without 1 mM H_2_O_2_ at room temperature for 1 min. The reactions were then stopped and washed with quenchers (10 mM sodium azide, 10 mM sodium ascorbate, and 5 mM Trolox) in PBS (with 1 mM CaCl_2_, 0.5 mM MgCl_2_). Cells were then harvested and lysed by 1 ml lysis buffer (50 mM Tris–HCl pH 8, 150 mM NaCl, 1% Triton-X 100, 2 mM EDTA pH 8) containing protease inhibitors and quenchers. 50 ul of streptavidin agarose beads (GE Health, 17511301) slurry was washed and incubated with cell lysates on rotation at room temperature for 1 h. The beads were subsequently washed with radioimmunoprecipitation assay buffer, 1 M KCl, 0.1 M Na_2_CO_2_, 2M urea in 10 mM Tris–HCl pH 8. After a final wash with radioimmunoprecipitation assay buffer, beads were eluted with 200 mM 1,4-DTT and 10 mM biotin with 4× lithium dodecyl sulfate buffer (Thermo Fisher Scientific: NP0007) for 10 min at 95 °C. Cells without H_2_O_2_ exposure were served as negative control. The streptavidin pull-down lysates and input were analyzed by Western blotting.

### Click-iT EdU flow cytometry

Cells were first incubated with 10 μM EdU in cultured medium at 37 °C. After 30 min, cells were washed, trypsinized, harvested, and processed following the manufacturer's instructions (Life technologies, C10419). Samples were then analyzed using BD Celesta Cell Analyzer.

### RNA-seq data analysis

Raw RNA-seq data in FASTQ format were aligned to mm10 reference genome using bowtie2 (61). The Sequence Alignment Map (SAM) file was converted to a Binary Alignment Map (BAM) file and then sorted by Samtools. Alignments with MAPQ score smaller than 30 were skipped. HTSeq (62) was used to calculate the number of mapped reads to each gene. The count matrix was imported to DESeq2 (63) for normalization, visualization, and analysis of differential gene expression. Empirical Bayes shrinkage estimators (64) were applied to shrink the limit fold change. The shrinkage of effect size was then used for visualization and ranking of genes. The significantly differentially expressed genes were identified by two thresholds (1): the absolute value of log2 fold change (LFC) greater than 0.5 or 1 (2); the adjust *p*-value (*p*-adj) less than 0.05. These differentially expressed genes were used for further Gene Ontology term enrichment analysis by R package clusterProfiler (65).

We used the method below to calculate the gene rescue capability (GRC) for MT194 and MT486 in Figure 3*I*. For each individual gene that was affected by NELFB knockout (EV-Cre *versus* EV-GFP) and restored by WT NELFB (WT-Cre *versus* EV-Cre), we defined them as NELFB-dependent gene. The relative restoration for this gene was set as 100% in WT-Cre and 0 in EV-Cre. To calculate the GRC, the relative restoration of this gene in either MT194-Cre or MT486-Cre was calculated and normalized with the relative restoration in WT-Cre, using the following formula:

$$GRC=\frac{{LFC}_{MT}-{LFC}_{NELFB}}{{LFC}_{WT\;NELFB}-{LFC}_{NELFB}}$$

The GRC is calculated for all NELFB-dependent genes in each MT. Next, we draw a diagram to present the entire distribution of the GRC from 0% to 100%.

### ChIP-seq data analysis

Raw ChIP-seq data in FASTQ format was aligned to mm10 reference genome using bowtie2 (61). The SAM file was converted to a BAM file and then sorted by Samtools. Alignments with MAPQ score smaller than 30 were skipped. The coverage (the number of reads per bin) track was generated by deepTools (66). The size of each bin was set at 1 bp and the coverage was normalized by Reads Per Kilobase per Million mapped reads. Scores for each gene were calculated by computeMatrix command. The gene bodies were scaled to 3 kb, and the region between 1 kb upstream to TSS and 1.5 kb downstream of transcription end sites were included when calculating the average reads coverage per genome regions.

### TR calculation

The locations for TSS regions (TSSRs, 350 bp) and GB regions (3000 bp) were located from the GRCm38 annotation file. The TSSR, GB region, and associated gene information were stored in bed files separately. Next, the read counts were calculated from BAM files and only the reads with MAPQ score greater than 30 were chosen. The read counts were further normalized if the input file was available. Lastly, minimum ChIP signal at TSSR was set as 0.001 to filter the weak peaks and the TR was calculated based on following formula (67):

$$TR=\frac{ReadCount\left(TSSR\right)/L1}{ReadCount\left(GBR\right)/L2}$$

where L1 and L2 are the lengths of TSS region and gene bodies region, respectively.

### DepMap data and Gene Ontology analysis

The public available database is from Cancer Dependency Project (https://depmap.org/portal/). The RNA expression data 21Q4 or 22Q1 were used for gene coexpression analysis. We obtained the top 1000 coexpressing genes from 1378 cell lines for NELFA, NELFB, NELFC, and NELFE, respectively. A Venn diagram was generated using UpSet Plot package to analyze the overlapping of coexpressing genes for different NELF subunits. Gene Ontology analysis was performed using web-based tool g:Profiler for top 1000 coexpressing genes of NELFB or coexpressing genes that are specific for each NELF subunit.

### Statistics and reproducibility

The results presented are representative of two or three independent experiments, as indicated for each graph in the figure legends. Student’s *t* tests were carried out using GraphPad Prisma 8 to calculate significance. Regression coefficients were calculated in Microsoft Excel. Results are expressed as mean ± SD unless indicated otherwise.

## Data availability

Source data are provided with this manuscript. All other data supporting the findings of this study are available from the corresponding author upon request. Sequencing datasets were deposited to NCBI Omnibus database and the accession number are GEO Submission (GSE205388) and GEO Submission (GSE205504).

## Supporting information

This article contains supporting information.

## Conflict of interest

The authors declare that they have no conflicts of interest with the contents of this article.

## Supporting information

Supplemental Tables

Supplemental Videos
