# Supplementary material for: An essential signaling function of cytoplasmic NELFB is independent of RNA polymerase II pausing
Source: J Biol Chem. 2023 Sep 17;299(11):105259. doi: 10.1016/j.jbc.2023.105259 (PMC10591015; doi:10.1016/j.jbc.2023.105259)
Supplement: Supplemental Figures [file mmc1.pdf]

Figure S1. Integrity of the NELF Complex is not Required for NELFB's Pro-survival Function

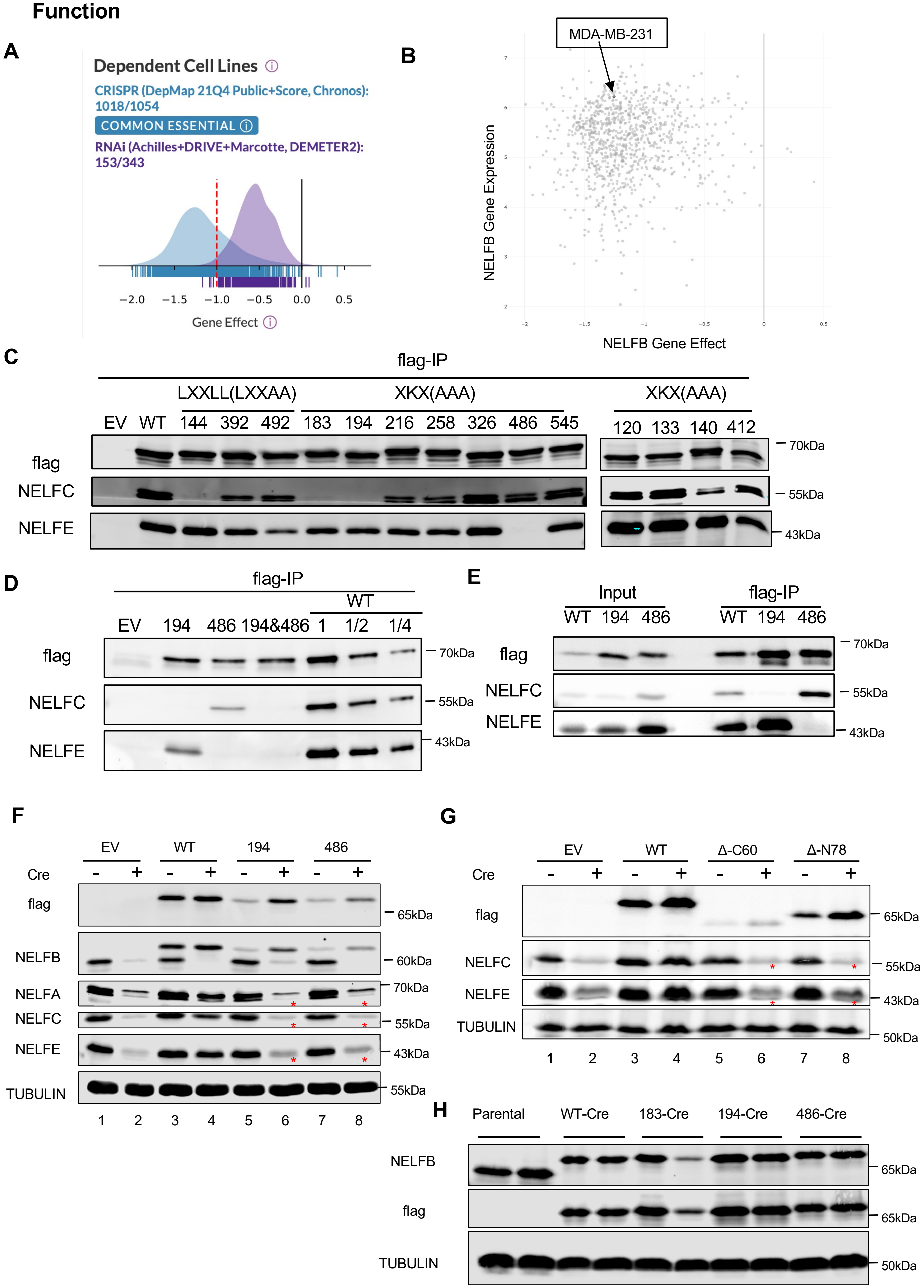

Figure S1. Integrity of the NELF Complex is not Required for NELFB's Pro-survival Function

(**A**) Graph from the Cancer Dependency project. Data from DepMap (21Q4 Public+Score, Chronos) indicate that NELFB is essential for 1,018 out of 1,054 cell lines. (**B**) Scatter diagram from the Cancer Dependency project shows the relationship between NELFB gene effects and NELFB gene expression in 1,054 cell lines. CRISPR data (21Q4 Public+Score, Chronos) and expression data (21Q4 Public) were used and MDA-MB-231 cell line is highlighted. No significant correlation is observed (Pearson: 0.018, p-val: 0.565). (**C**) Co-IP of 293T cells transfected with EV, flag-tagged WT and mutant NELFB. Representative image from 2 independent experiments is shown. (**D**) Co-IP of MEFs stably expressing EV, WT or NELFB mutants. Representative image from 2 independent experiments is shown. (**E**) Co-IP of MEF clones with ectopic WT or NELFB mutants without endogenous NELFB. Representative image 2 independent experiments is shown. (**F, G**) Stable *Nelfb*$^{f/-}$ cell lines were established with overexpression of EV, WT, or various mutants. Upon Cre-mediated floxed *Nelfb* deletion, NELF subunits were analyzed by Western blotting. Representative result from 3 independent experiments is shown. (**H**) WT and NELFB mutant MEF clones upon *Nelfb* deletion were analyzed by western blotting. Experiment was performed using 2 independent clones for each line.

Figure S2. C-terminal deletion of NELFB in 231 cells destabilizes NELF complex without affecting cell survival

A

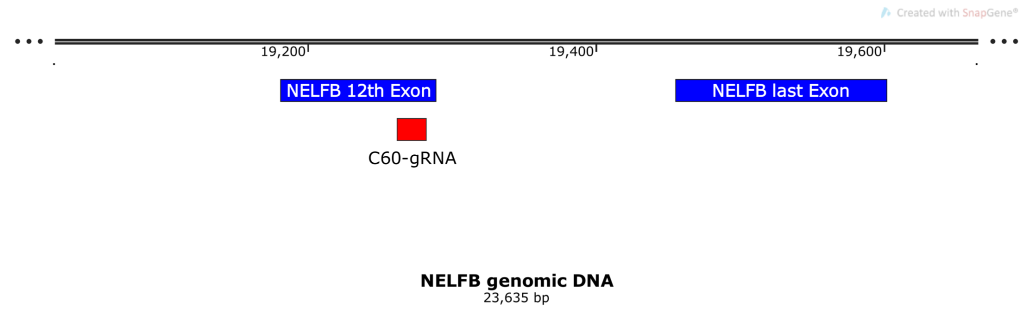

B

Alignment statistics of AAs from Last Two Exons between Human and Mouse NELFB					
Score	Expect	Method	Identities	Positives	Gaps
125 bits(313)	2e-44	Compositional matrix adjust.	79/85(93%)	81/85(95%)	0/85(0%)

```
NELFB [Homo sapiens]  1   RKENVHRHALRLLIHLHPRVAPSKLEALQKALEPTGQSGEAVKELYSQLGEKLEQLDHRK  60
                          RKENVHRH LRLL+HLH RVAPSKLEALQKALEPTGQSGEAVKELYSQLGEKLEQLDHRK
NELFB [Mus musculus]  1   RKENVHRHVLRLLLHLHARVAPSKLEALQKALEPTGQSGEAVKELYSQLGEKLEQLDHRK  60
NELFB [Homo sapiens]  61  PSPAQAAETPALELPLPSVPAPAPL  85
                          PSP QAAETPAL+LPLPSVPAPA L
NELFB [Mus musculus]  61  PSPTQAAETPALDLPLPSVPAPATL  85
```

C

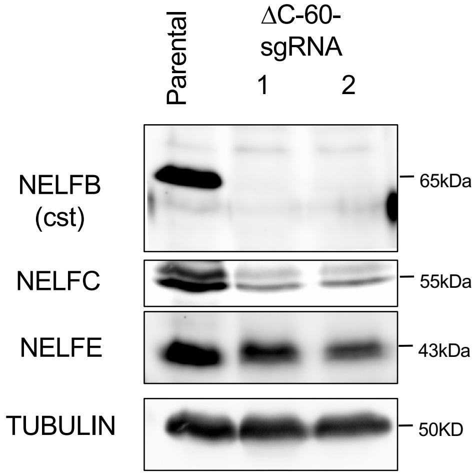

D

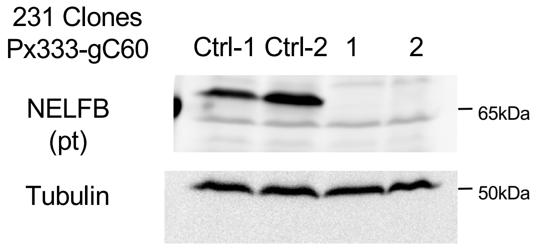

E

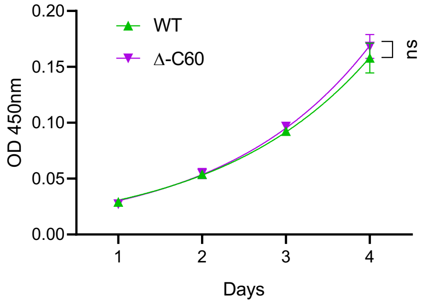

Figure S2. C-terminal deletion of NELFB in MDA-MB-231 cells destabilizes NELF complex without affecting cell survival

(**A**) Diagram of CRISPR-mediated gene editing targeting NELFB exon 12 for C-terminal deletion in MDA-MB-231 cells. (**B**) Alignment analysis for the C-terminal Amino Acid (AA) sequences (last two exons) between human and mouse NELFB using Basic Local Alignment Search Tool (BLAST) from NCBI. (**C**) Western blotting of NELFB (Cell Signaling, 14894), NELFC, and NELFE protein expression in MDA-MB-231 Δ-C60 clones. Experiment was performed using two independent clones. (**D**) Western blotting of NELFB in MDA-MB-231 Δ-C60 clones using a different NELFB primary antibody (Proteintech, 16418-1-AP). Experiment was performed using two independent clones. (**E**) Cell growth curves for MDA-MB-231 WT and ΔC-60 clones. Result was average of 2 independent clones.

Figure S3. NELFB-dependent Pol II pausing and cell proliferation are functionally separable

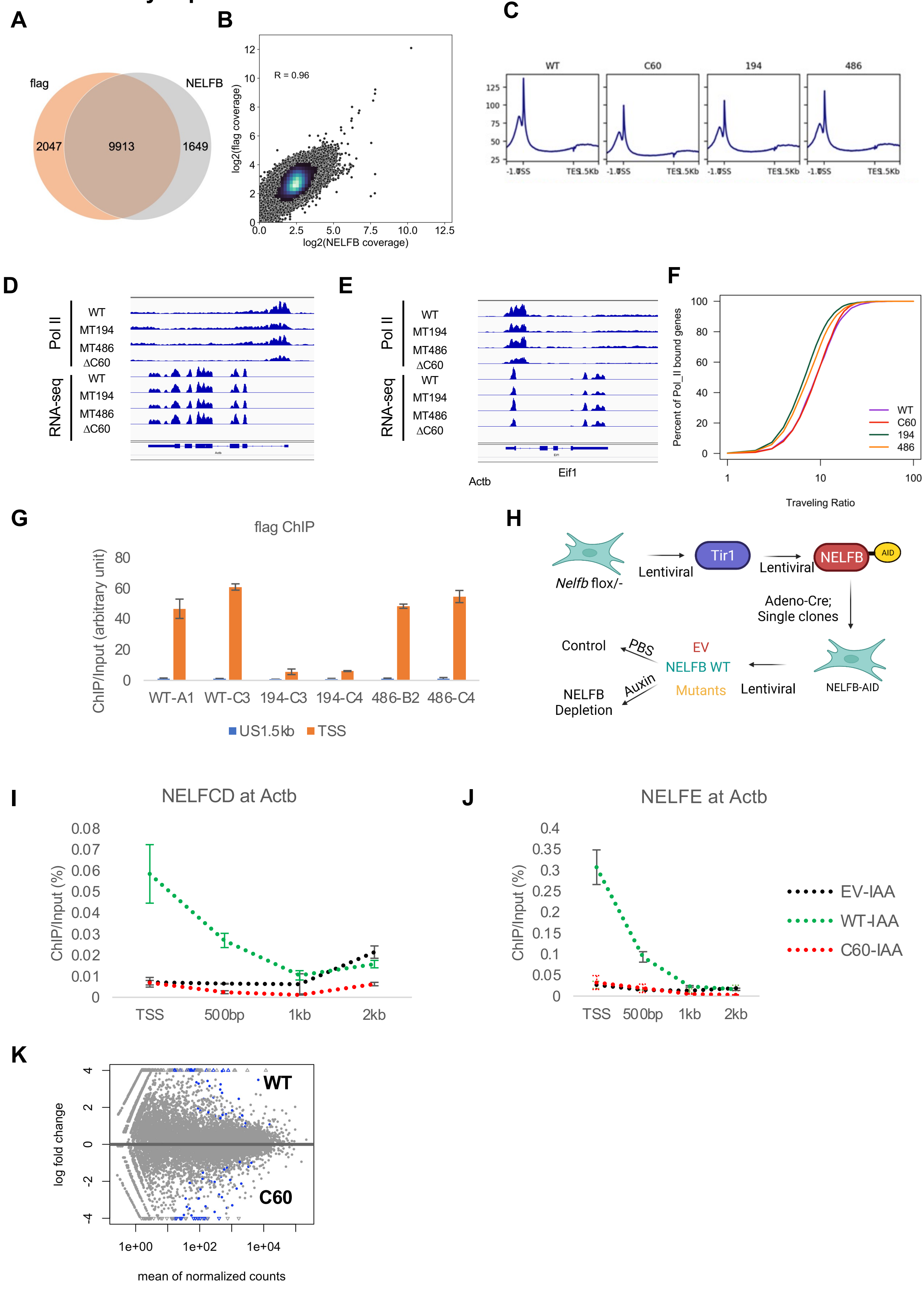

Figure S3. NELFB-dependent Pol II pausing and cell proliferation are functionally separable.

(**A**) Venn diagram shows the overlapping binding sites between flag ChIP-seq (in flag-WT-NELFB MEFs) and published NELFB ChIP-seq. (**B**) Diagram shows the correlation of flag ChIP-seq (in flag-WT-NELFB MEFs) and published NELFB ChIP-seq. (**C**) Averaged metagene profiles of pol II chromatin occupancy in WT, MT194, MT486 and Δ-C60 cells. ChIP-seq experiments were performed with two independent biological replicates. The analyzed region is from −1kb upstream of transcription start sites (TSS) to +1.5kb downstream of transcription end sites (TES). (**D, E**) Representative IGV profiles at two NELFB bound genes *Actb* (D) and *Eif1* (E). Pol II ChIP-seq was average of 2 independent clones. RNA-seq was average of three independent clones. (**F**) Cumulative curves of Pol II traveling ratios for WT, MT194, MT486 and Δ-C60 clones. (**G**) ChIP-qPCR that measured chromatin occupancy of WT NELFB, MT194 and MT486 at the *Actb* TSS and 1.5kb upstream of TSS. (**H**) Schematic illustration of generating NELFB-AID cells and testing NELFB mutants' activities using NELFB-AID cells. (**I, J**) ChIP-qPCR that measures chromatin occupancy of NELFC (H) or NELFE (I) at the *Actb* TSS, and 500bp, 1kb or 2kb downstream of TSS. NELFB-AID Cells with EV, WT and ΔC-60 were treated with IAA for 6 hours. Two independent experiments were performed and the averages were calculated. (**K**) MA plot shows RNA-seq from WT and Δ-C60 clones with three independent biological replicates.

Figure S4. NELFB-dependent transcriptome does not entirely depend on an intact TSS-bound NELF complex

A

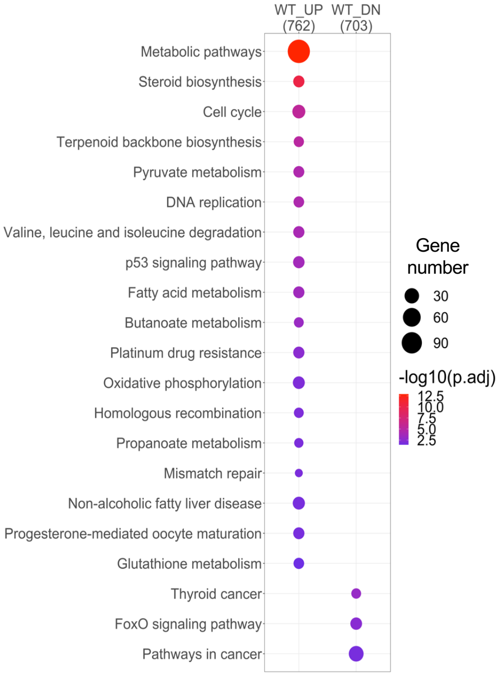

B

Percentage of genes restored by WT or NELFB mutants			
EV	WT	MT194	MT486
200 (NELFB-downregulated)	99.5%	68.0%	75.0%
177 (NELFB-upregulated)	97.1%	67.8%	82.5%
377 (total)	98.4%	67.9%	78.5%

C

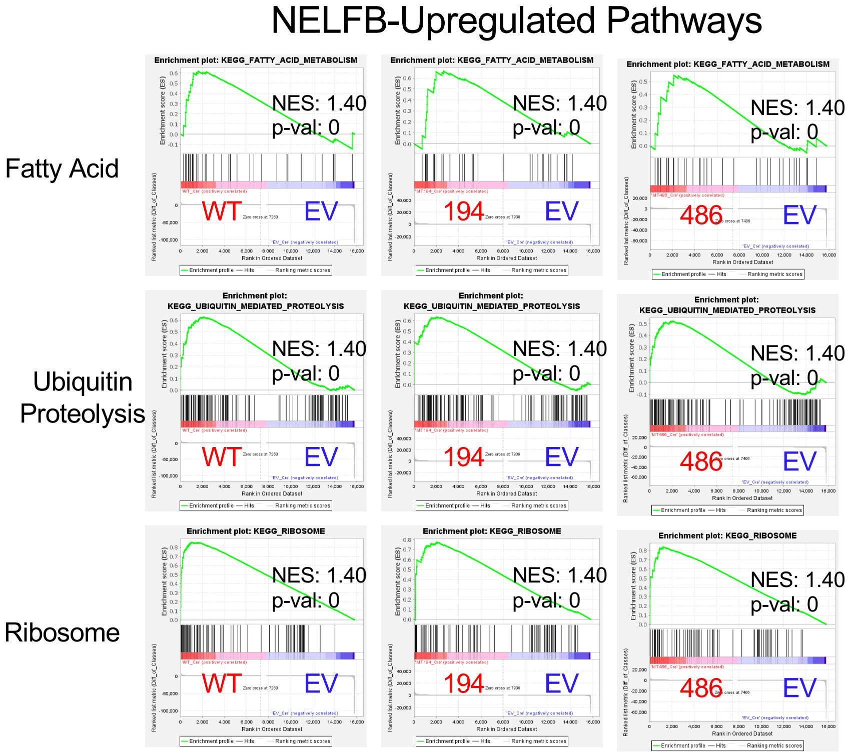

D

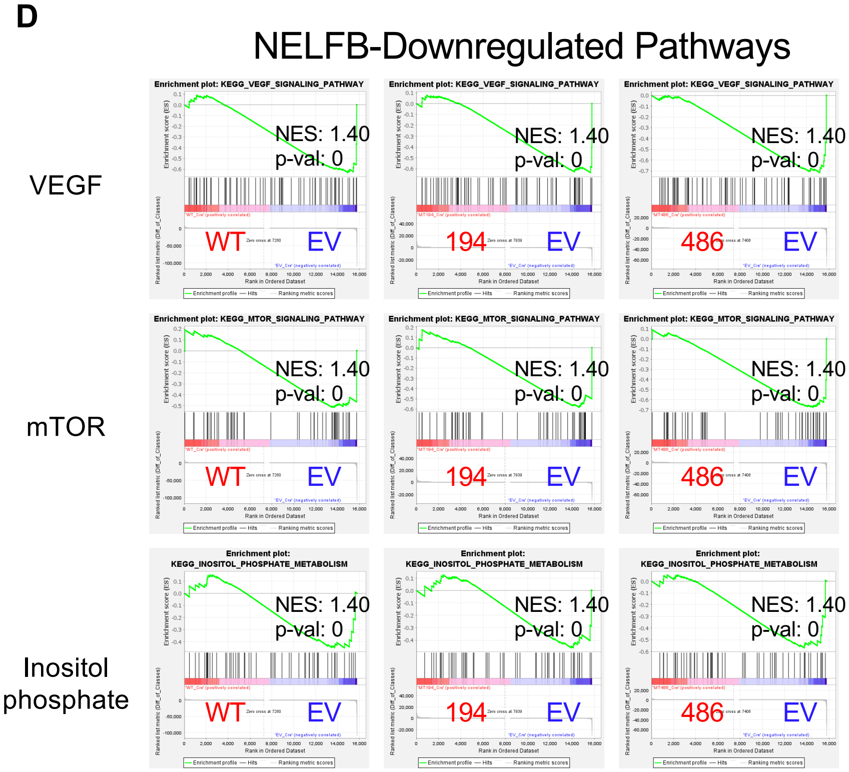

Figure S4. NELFB-dependent transcriptome does not entirely rely on an intact TSS-bound NELF complex

(**A**) Kyoto Encyclopedia of Genes and Genomes (KEGG) pathway enrichment analysis of NELFB-upregulated (762) and -downregulated genes 703), based on RNA-seq data from *Nelfb*$^{f/-}$ cells with EV or ectopic WT, treated with Cre-expressing adenovirus. The number of enriched genes is shown with different sizes and the p-val of the enrichment indicated with different colors. (**B**) Table showing the percentage of NELFB-downregulated or -upregulated genes (Fig. 3A) restored by WT, MT194 or MT486. 200 genes were NELFB-downregulated and 177 genes were NELFB-upregulated using a cut off of abs (log 2FoldChange) > 1 and adjusted p-val < 0.05. (**C, D**) GSEA of RNA-seq data between EV and WT, or EV and mutant cells on NELF-upregulated (C) and -upregulated (D) genes.

Figure S5. NELFC binding-deficient mutants of NELFB are retained in the cytoplasm NELFB

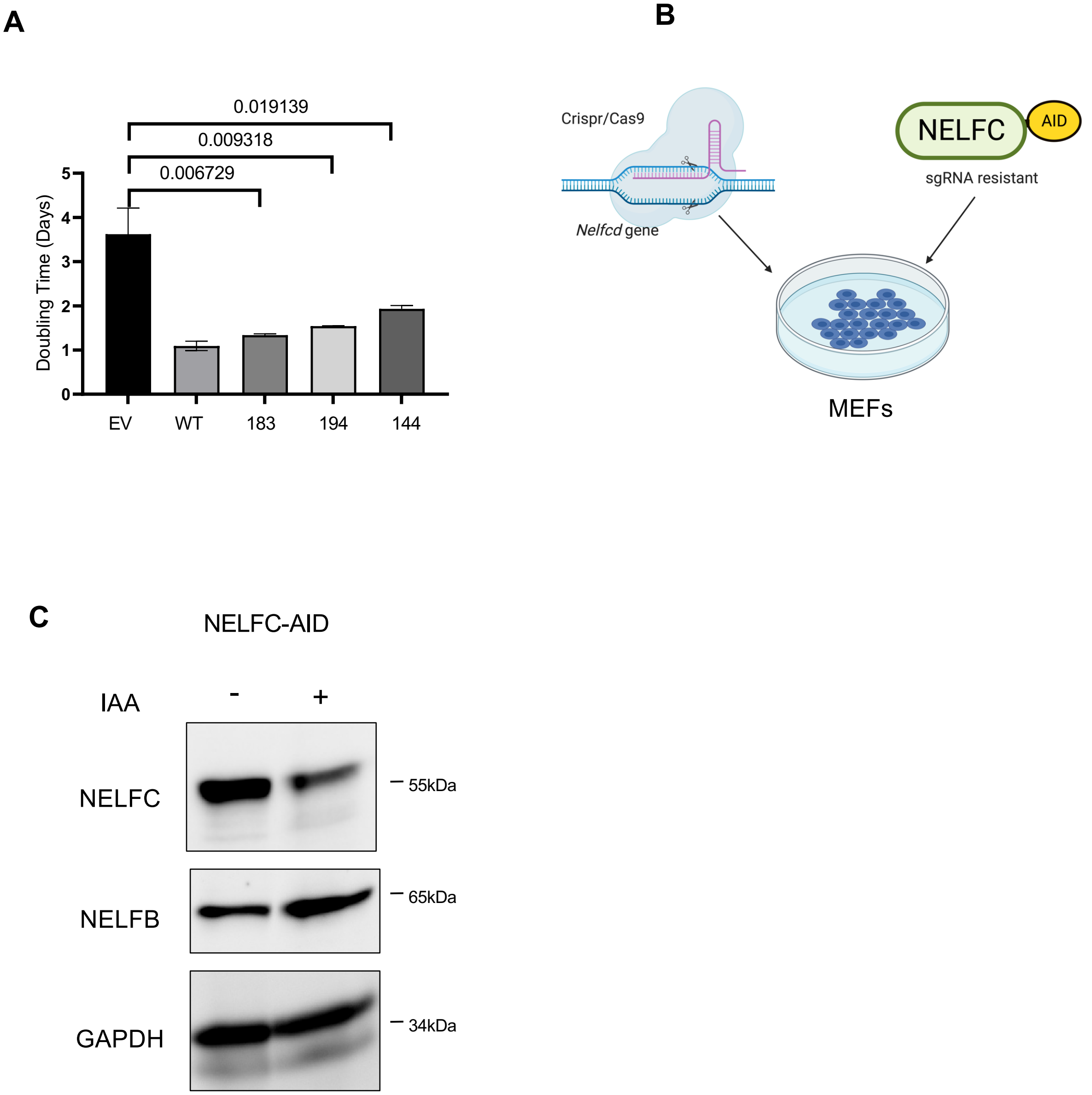

Figure S5. NELFC binding-deficient mutants of NELFB are retained in the cytoplasm.

(**A**) NELFB-AID stable cell lines that expressed EV, WT, MT183, and MT194 were treated with either PBS or IAA continuously. Cell proliferation was measured for 4 consecutive days. Doubling time was calculated using nonlinear regression of exponential (Malthusian) growth model. Statistical analysis was performed using multiple t test to compare mutant with EV, and p-val was indicated to the corresponding mutants. The results were average of 2 independent experiments. (**B**) Schematic illustration of NELFC-AID MEFs. (**C**) Western blotting of NELFC and NELFB in NELFC-AID cells after IAA treatment for 3 days. Representative result from three biological repeats was shown.

Figure S6. NELFB is physically associated with signaling molecules

A

Cor	PValue	QValue	label	numCellLines
0.593142	1.02E-131	8.66E-130	ABL1	1378
0.579748	1.49E-124	1.01E-122	MAPK1	1378
0.578505	6.61E-124	4.40E-122	RELA	1378
0.571219	3.66E-120	2.13E-118	AKT2	1378
0.564831	5.89E-117	3.08E-115	PAK4	1378
0.56394	1.63E-116	8.37E-115	AKT1	1378
0.558632	6.53E-114	3.11E-112	PDPK1	1378
0.547061	2.14E-108	8.41E-107	RAF1	1378
0.546105	5.97E-108	2.31E-106	PAK2	1378
0.525575	1.04E-98	3.07E-97	GRB2	1378
0.523569	7.69E-98	2.21E-96	SH2B1	1378
0.521926	3.92E-97	1.10E-95	NFATC3	1378
0.514158	7.71E-94	1.97E-92	CAMKK2	1378
0.513166	2.00E-93	5.04E-92	PIK3R2	1378
0.505711	2.36E-90	5.39E-89	MAPK14	1378
0.502526	4.59E-89	1.01E-87	IKBKG	1378
0.502488	4.76E-89	1.04E-87	MTOR	1378

B

C

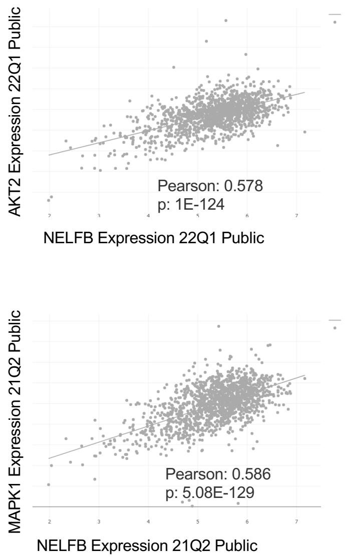

D

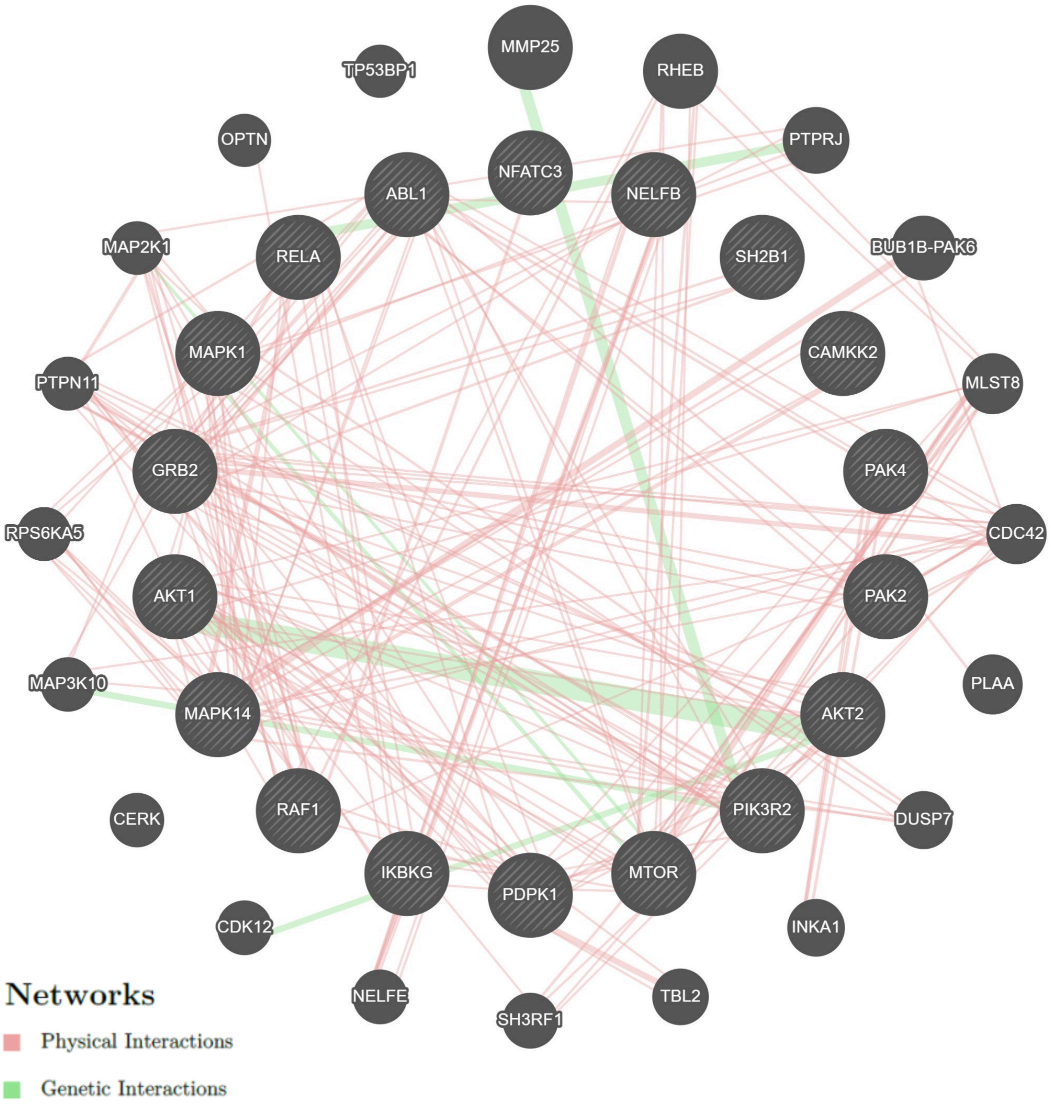

Figure S6. NELFB is Physically Associated with Signaling Molecules.

(**A**) Gene expression correlation between NELFB and 17 signaling molecules. The data was from DepMap Data Expression 21Q4 Public. (**B, C**) Correlation between NELF and AKT2 (B) or MAPK1 (C), using DepMap Data Expression 22Q1 Public. (**D**) Network interaction graph illustrating physical interactions (pink) and genetic interactions (green) for NELFB and the co-expressing signaling genes using GeneMANIA (www.genemania.org).

Figure S7. NELFB supports pro-survival signal transduction

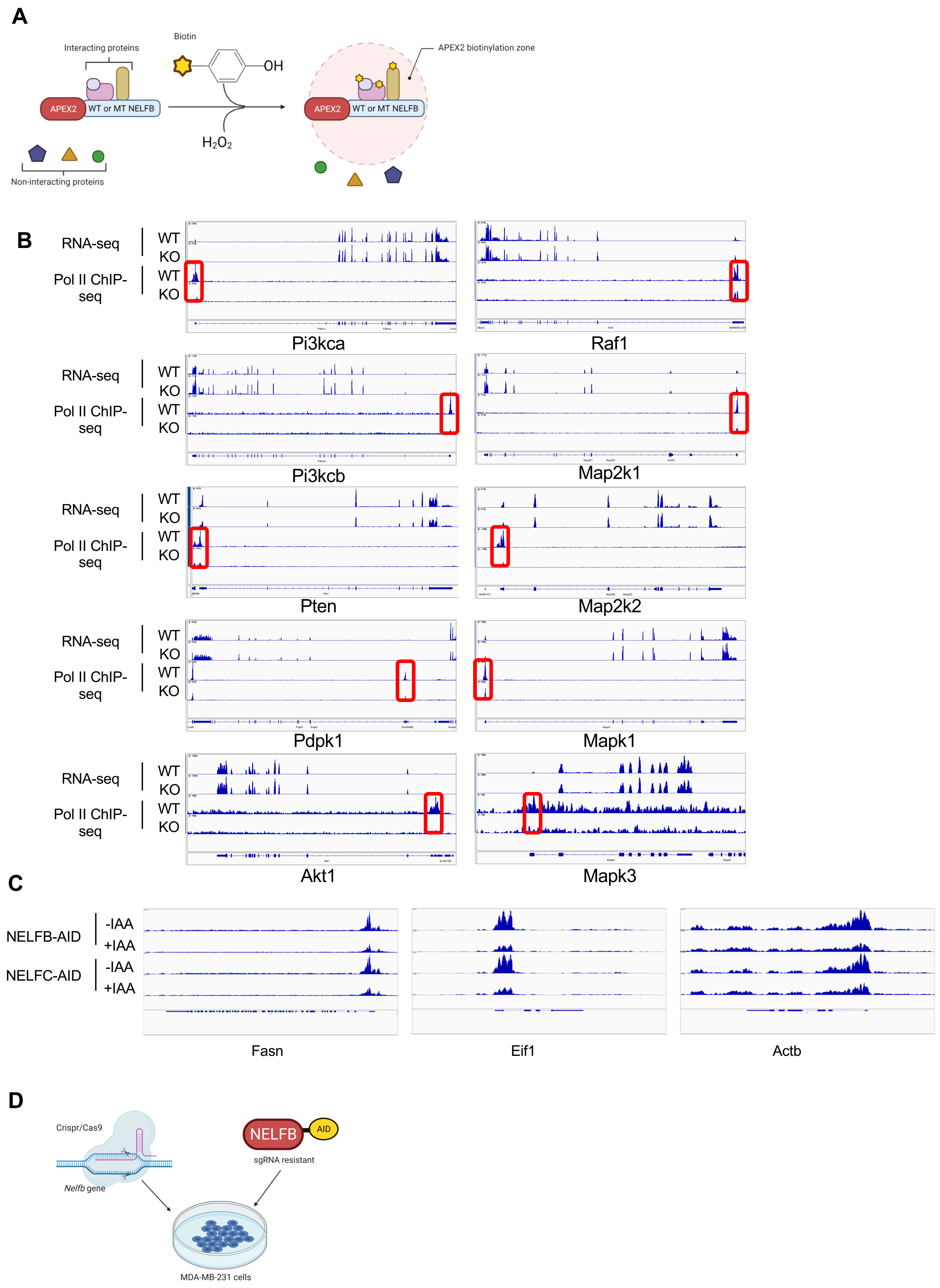

Figure S7. NELFB supports pro-survival signal transduction.

(**A**) Apex2-based proximity labeling. (**B**) IGV profiles showing RNA-seq and Pol II ChIP-seq data from WT and NELFB KO MEFs for genes directly involved in PI3K/AKT and MEK/ERK pathways. (**C**) IGV profiles showing Pol II ChIP-seq data from NELFB-AID and NELFC-AID cells following treatment of PBS or 0.5mM IAA for 6 hours. (**D**) Schematic illustration of generation of NELFB-AID-expressing MDA-MB-231 cells.

Figure S8. AKT1's effects on Pol II occupancy in NELFB depleted cells

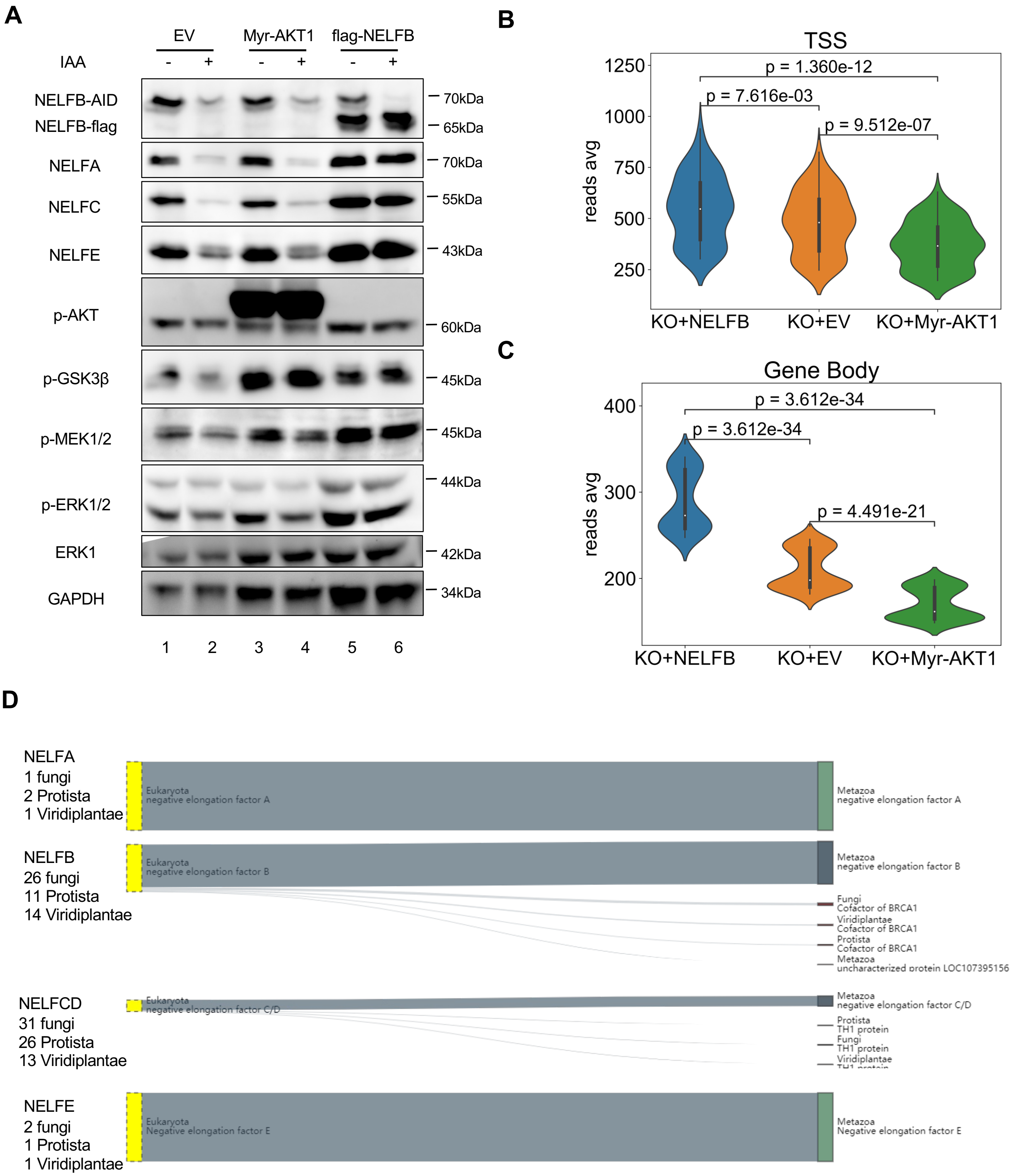

E

Protein Name	Position	Sequence	Score
NP_067368 NELFB [Mus musculus]	145-159	ENGVLLPSLQSALPF	0.364
NP_056271.3 NELFB [Homo sapiens]	137-151	ENGVLLPSLQSALPF	0.364
NP_067368 NELFB [Mus musculus]	146-160	NGVLLPSLQSALPFL	0.367
NP_056271.3 NELFB [Homo sapiens]	138-152	NGVLLPSLQSALPFL	0.367
NP_067368 NELFB [Mus musculus]	198-212	RYKKLEDLLEKSFSL	0.442
NP_056271.3 NELFB [Homo sapiens]	190-204	RYKKLEDLLEKSFSL	0.442
NP_067368 NELFB [Mus musculus]	442-456	RDSPDLLLLLRLLAL	0.539
NP_056271.3 NELFB [Homo sapiens]	434-448	RDSPDLLLLLRLLAL	0.539

Figure S8. AKT1's effects on Pol II occupancy in NELFB-depleted cells.

(**A**) Stable NELFB-AID MEF cell lines expressing EV, Myr-AKT1 or flag-NELFB were treated with IAA and cell lysates were analyzed by western blotting. Representative images from 3 independent experiments are shown. (**B, C**) NELFB-AID MEFs overexpressing EV, Myr-AKT1 or NELFB were treated with IAA for 6 hours and Pol II occupancy in these cell lines was analyzed by ChIP-seq. Violin plot distributions of Pol II ChIP-seq reads at the TSS (−0.5 to +0.5kb, A) and GB (+0.5 to +2.5 kb, B) in EV, Myr-AKT1 or NELFB cells. Each cell line had three independent repeats. (**D**) Group hierarchies of NELF subunits at Eukaryota level. The graphs were generated using OrthoDB v10.1. (**E**) Prediction of Nuclear Export Signals (NES) for mouse and human NELFB using LocNES algorithm.
